# *APOE*‐stratified Proteomic and Metabolomic Analysis Reveals Mitochondrial Dysfunction Inflammation and Lipid Dysregulation in Alzheimer's Disease

**DOI:** 10.1002/advs.202513872

**Published:** 2026-02-05

**Authors:** Fuhai Li, Yike Chen, Daniel Western, Muhammad Ali, Menghan Liu, Katherine Gong, Ying Xu, Joseph Lowery, David M. Holtzman, Chloe Robins, John D. Eicher, Yen‐Ning Huang, ShiWei Liu, Tamina Park, Andrew J. Saykin, Kwangsik Nho, Mahdi Moqri, Richard C. Mohs, Amelia Farinas, Patricia Moran‐Losada, Hamilton Se‐Hwee Oh, Tony Wyss‐Coray, Carlos Cruchaga

**Affiliations:** ^1^ Institute For Informatics (I2) Washington University in St. Louis School of Medicine St. Louis Missouri USA; ^2^ Department of Pediatrics Washington University in St. Louis School of Medicine St. Louis Missouri USA; ^3^ Department of Psychiatry Washington University in St. Louis School of Medicine St. Louis Missouri USA; ^4^ NeuroGenomics and Informatics Washington University in St. Louis School of Medicine St. Louis Missouri USA; ^5^ Department of Neurology Knight Alzheimer's Disease Research Center Hope Center for Neurological Disorders . St. Louis Missouri USA; ^6^ Human Genetics & Genomics Research Technologies GSK Cambridge MA USA; ^7^ Center For Neuroimaging Indiana Alzheimer's Disease Research Center Department of Radiology and Imaging Sciences Indiana University School of Medicine Indianapolis IN USA; ^8^ Division of Genetics Department of Medicine Brigham and Women's Hospital Harvard Medical School Boston Massachusetts USA; ^9^ Global Alzheimer's Platform Foundation Washington DC USA; ^10^ The Phil and Penny Knight Initiative for Brain Resilience Stanford University Stanford California USA; ^11^ Wu Tsai Neurosciences Institute Stanford University Stanford California USA; ^12^ Icahn School of Medicine at Mount Sinai New York New York USA; ^13^ Department of Neurology and Neurological Sciences Stanford University School of Medicine Stanford California USA

**Keywords:** APOE, alzheimer, proteomics, metabolomics

## Abstract

*Apolipoprotein E (APOE*) ε4 is the strongest genetic risk factor for Alzheimer's disease (AD). However, it is known that other pathways independent of *APOE* also play a role in AD. Disentangling *APOE*‐dependent and independent effects is instrumental for understanding the biology of AD. We conducted an *APOE*‐stratified multi‐omic analysis in multiple large datasets to identify AD‐associated plasma proteins and metabolites. More than 64% of the identified proteins were not found in non‐*APOE* stratified studies, and 17% of the proteins showed *APOE*‐specific trends. Mitochondrial dysfunction was associated in AD independently of *APOE* and was accompanied by disruptions in glucose and lipid metabolism and cell death and increased in inflammatory signaling activation. Lipid upregulation was found in AD cases when compared with controls with the same *APOE* genotype, indicating that additional factors beyond *APOE* affect lipid regulation and AD risk. These findings may be informative in guiding the development of effective medications for AD.

## Graphical abstract

A large‐scale multiomic dataset (proteomic and metabolomic) comprising 3,060 plasma samples were analyzed to identify proteins, metabolites, pathways, and protein‐associated drugs linked to Alzheimer’s Disease (AD) independently of apolipoprotein E (APOE). AD was associated with a distinct molecular signature that captures. mitochondrial damage and lipid accumulation, which exacerbate inflammation and AD pathology independently of APOE genotype.xx xx

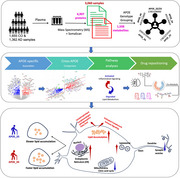

## 1 Introduction

About 6.5 million people are living with Alzheimer's disease (AD) in the US. The healthcare cost of AD is extreme, currently estimated at ∼$321 billion and expected to increase to $1 trillion by 2050 [1], demonstrating the importance of understanding AD development and etiology. Though more than 74 genes/loci have been associated with AD [2, 3, 4], including *APOE* [5], *TREM2* [6, 7], *and MS4A4A* [8, 9], the underlying pathogenesis and signaling pathways that lead to neurodegeneration remain unclear. The main pathological features of AD are the accumulation of amyloid plaques and neurofibrillary tangles in the brain, which are composed of misfolded proteins called amyloid‐beta (Aβ) and tau, respectively [10]. Recently, immunotherapies targeting Aβ, like aducanumab, lecanemab [11], and donanemab [12, 13] have shown promising effects including slowing of disease progression and cognitive decline in patients with mild dementia due to AD. However, there are no treatments that can prevent AD [1, 14]. While tau and the immune response are also promising targets, there may be additional mechanisms that can be discovered to target AD [15, 16, 17].

Among the AD‐associated genetic markers, the *APOE* ε4 allele is the strongest genetic risk factor for sporadic AD [17, 18]. There are three common haplotypes for *APOE*: *APOE* ε2*, APOE* ε3, and *APOE* ε4. There are six possible *APOE* genotypes, that is, *APOE* ε2/ε2 (22), ε2/ε3 (23), ε2/ε4 (24), ε3/ε3 (33), ε3/ε4 (34), and ε4/ε4 (44). The *APOE* ε2 allele is associated with lower AD risk and is a longevity biomarker [19]. The *APOE* heterozygous (ε3/ε4) and homozygous (ε4/ε4) genotypes are associated with up to 3 and 15‐fold increases in AD risk, respectively, compared with the *APOE* ε3/ε3 genotype [17]. *APOE* is involved in lipid metabolism and in the transport of cholesterol and other fats through the body. Dysfunction or inefficiency of lipid transport or of cholesterol and fat metabolic processes might cause lipid accumulation particularly in the CNS, a process that has recently been linked to *APOE* and AD pathogenesis [20]. However, as AD cases are highly enriched for *APOE* ε4 alleles, it is challenging to disentangle the pathologic events that lead to AD that are dependent on and independent of *APOE*.

Proteomic and metabolomic studies of AD are also important to understand and identify novel molecular biomarkers, pathways, and therapeutic targets [21, 22, 23, 24, 25, 26]. Many studies have been conducted to investigate the proteomic changes in different brain regions, cerebrospinal fluid (CSF), or blood plasma from AD patients or animal models. For example, in a recent CSF proteomic study, 125 proteins were identified as novel biomarkers associated with early, middle, and presymptomatic stages of Autosomal Dominant Alzheimer's disease (ADAD) [24]. In another study using the same proteomic platform, 2,173 dysregulated proteins were identified and validated in independent cohorts as being associated with sporadic AD. These proteins are involved in neuronal death, tau phosphorylation, and microglia‐neuron interactions, and can be used as biomarkers to predict specific AD stages [25]. Moreover, another study analyzing brain, CSF, and plasma samples of AD and control groups identified protein signatures (8 brain, 40 CSF, and 9 plasma protein biomarkers) that can distinguish sporadic and genetic AD [22]. A smaller proteomic study (271 proteins analyzed) in ∼1,000 dorsolateral prefrontal cortex (DLPFC) tissues from control, asymptomatic AD (AsymAD) and AD brains identified disease‐specific co‐expression modules [27]. Studies using protein co‐expression analysis identified several proteins in the matrisome pathway to be associated with AD [23]. These analyses have demonstrated the potential of proteomics to advance our understanding of AD biology and pathology and to discover new biomarkers and potential therapeutic strategies for this devastating disease. At the same time, a metabolomic study found that insulin resistance is associated with AD [28]. Altered glucose and carbon metabolism [29] and energy metabolism [30] were also identified using proteomics and metabolomics in CSF. A landscape of metabolic brain alterations in AD that included 500 post‐mortem brain samples from the DLPFC of the Accelerating Medicine Partnership Program for Alzheimer's Disease (AMP‐AD) consortium identified 298 metabolites across pathways related to bioenergetics, cholesterol metabolism, neuroinflammation, and neurotransmitter imbalances, which indicated impaired osmoregulation as a potential AD mechanism and suggested that tau pathology, rather than Aβ pathology, primarily drives these metabolic changes [31].

As *APOE* is the major genetic risk factor for AD, and AD cases are highly enriched for *APOE* ε4 alleles while controls are enriched for ε3 or ε2, it has been difficult in previous studies to determine if the proteomic and metabolomic changes found in AD are driven by *APOE* or additional factors. Recently there have been several studies looking at *APOE*‐specific proteomic signatures of AD [32, 33]. In the first study, CSF proteomics were generated from 300 samples and protein co‐expression networks associated with AD were identified, including one highly associated with *APOE ε*4 which was involved in oxidant detoxification, mitogen‐associated protein kinase signaling, neddylation, and mitochondrial biology [32]. Another proteomic study identified 303 proteins associated with incident AD [33]. This study attempted to identify *APOE‐*independent signals by performing *APOE*‐ε4 interaction analysis, in which interaction and conditional terms for *APOE* were included in the model, but this is not equivalent to proper *APOE* genotype stratification. Of the AD‐associated proteins, 17 were driven by *APOE*, while 140 proteins were classified as *APOE*‐independent. Although these recent studies indicate that there may be proteins associated with AD that are dependent on and independent of *APOE*, one major limitation of the reported studies is that they were not able to fully disentangle the *APOE*‐dependent and *APOE‐*independent pathways. As a result, these studies were unable to elucidate the distinct biological mechanisms through which *APOE*‐dependent and *APOE*‐independent pathways contribute to AD pathogenesis. Therefore, large‐scale studies specifically designed to investigate individual *APOE* genotypes are needed.

To fully understand the pathological events associated with AD, it is instrumental to separate those that lead to disease independently of *APOE*. In this study, we present a systematic profiling and analysis of large‐scale plasma proteomics (6,907 aptamers) and metabolomics (1,508 features) using data from a total of 3,060 individuals (1,655 control and 1,362 AD), analyzing the association between each factor and AD status stratified by *APOE* genotype. We further replicate our results in five additional independent datasets including more than 50,000 samples. By comparing the proteins associated with AD in each *APOE*‐stratified analysis, we identified mitochondrial and lipid metabolic processes (linked to lipid accumulation) as the major signaling pathways associated with disease independently of *APOE*.

## 2 Results

### 2.1 Study Design

The primary goal of this study was to identify proteomic and metabolomic changes and their associated pathways in AD that are not driven by *APOE*. This can illuminate unknown pathogenic factors and guide the development of novel therapeutic strategies for AD prevention and treatment. To do this, instead of conditioning on *APOE* or identifying networks associated with *APOE* as done in previous studies, we performed proteomic and metabolomic analyses in each *APOE* genotype independently. We analyzed large‐scale plasma proteomics and metabolomics datasets from a large and well characterized cohort. Specifically, we generated and analyzed proteomics (6,907 features) and metabolomics (1508 features) from a total of 3,060 samples, comprising 1,655 control and 1,362 AD samples from the Knight‐ADRC cohort that covers all *APOE* genotypes (*APOE_2X* (22, 23 and 24): 89 AD/170 Control; *APOE_24*: 40/45; *APOE_33*: 535/778; *APOE_34*: 334/209, *APOE_44*:128/43; Tables S1 and S2, Figure S1).

To identify robust and reliable findings, we performed a four‐stage study design, including internal discovery (*n* = 1,381) and replication (*n* = 1,382), followed by meta‐analyses and then validation in external datasets. For each stage, proteomic and metabolomic data of AD cases and controls carrying the same *APOE* genotype were compared independently: *APOE_2x (22, 23 and 24), APOE_24*, *APOE_33*, *APOE_34*, *APOE_44*, and *APOE_4x* (34/44). Proteins and metabolites associated with AD status in each *APOE* genotype were identified by performing logistic regression adjusted by the age and sex covariates. The analytes identified in the discovery dataset were further tested in the replication dataset. Only those that were associated with AD in both discovery and replication datasets with the same direction (up‐/down‐regulated) and that passed FDR correction in the meta‐analysis for the entire study were considered significant. To further validate the findings, the proteins identified in the meta‐analysis were further validated in six independent datasets: The Global Neurodegeneration Proteomics Consortium (GNPC), Indiana‐ADRC (IADRC), Bio‐Hermes, UK Biobank, and the Stanford ADRC dataset, which includes both SomaScan and Olink platforms.

After the identification of the *APOE*‐independent proteins and metabolites associated with AD, we performed pathway enrichment, cell‐type enrichment analysis, and signaling network analysis to uncover the key dysfunctional signaling pathways and biological processes in AD. Moreover, we also compared the effect size of the identified proteins and metabolites across the *APOE* genotypes to identify *APOE* genotype‐specific and shared signals. We further conducted drug repositioning analysis by integrating drug target information derived from DrugBank [34, 35] and reverse gene signature information from Connectivity Map (CMAP) to identify potential therapeutic targets from these protein biomarkers and medications for potential AD prevention and treatment. These analyses identified the key signaling pathways and targets driving AD pathogenesis, which are novel and not reported in the existing non‐*APOE* stratified analyses.

### 2.2 *APOE*‐stratified Analysis Identifies Diverse Proteomic and Metabolomic Changes Associated With AD

**FIGURE 1:**
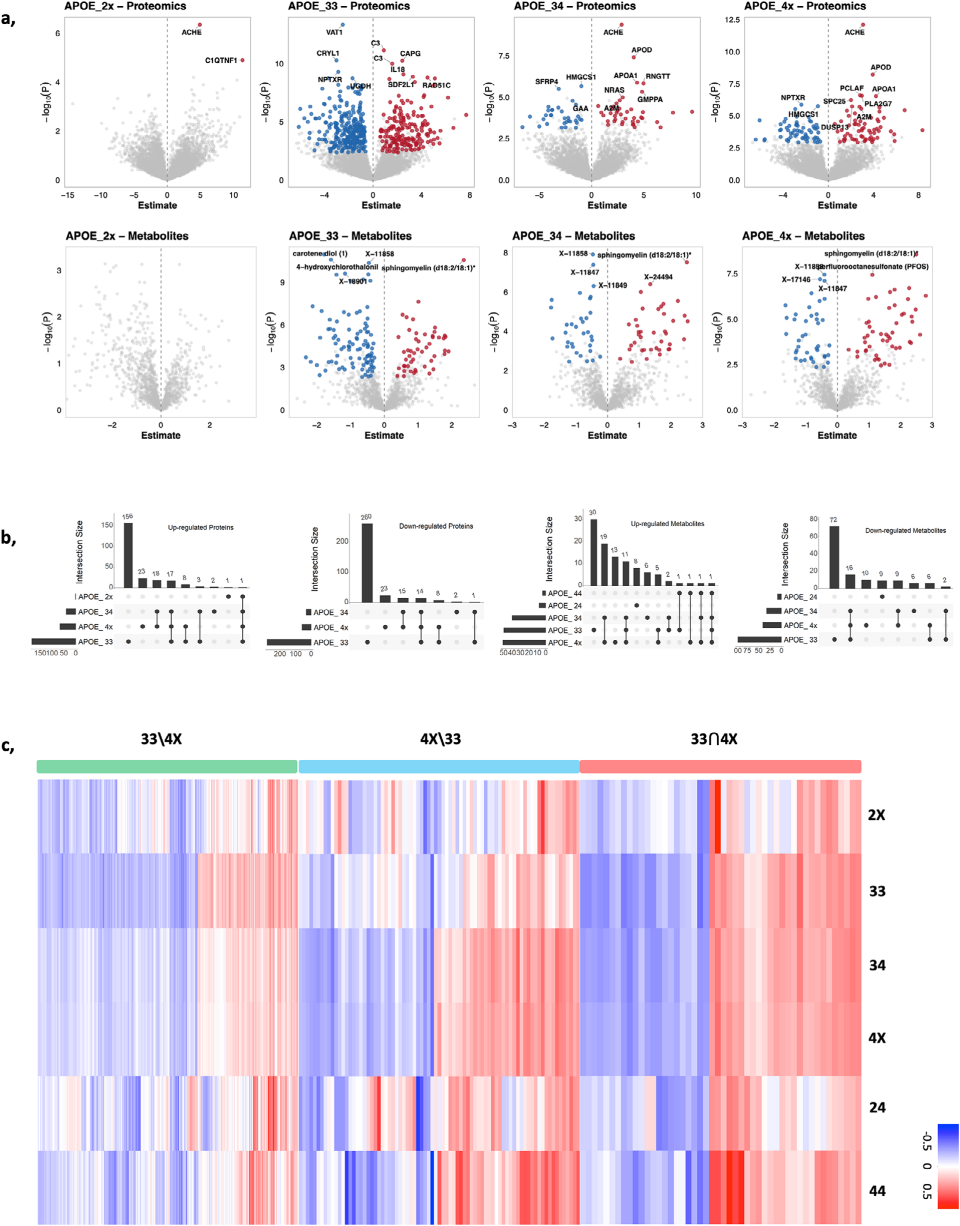
APOE stratified differential analysis identified diverse proteomic and metabolomic biomarkers. (a) Volcano‐plots of proteins and metabolites of *APOE*_2x, *APOE*_33, *APOE*_34 and *APOE*_4x. (b) UpSet plots of up‐ and down‐regulated proteins and metabolites across different *APOE* genotypes. (c) Heatmap plot of average aptamer abundance in each *APOE* genotype shows the signaling patterns of the differentially expressed aptamers across six *APOE* genotypes. There are three sets of aptamers used: 33⋂4X (48 aptamers selected in both *APOE*_33 and *APOE*_34), 4X∖33 (79 aptamers only selected in *APOE*_4X), and 33∖4X (420 aptamers only selected in *APOE*_33).

In the discovery dataset, we identified from 150 to 1,500 proteins and 80 to 350 metabolites associated with AD status for each *APOE* genotype (Figure 1, Table S3, Figure S2). The *APOE* genotypes 2x, 24, and 44 presented the lowest number of analytes associated with AD due to low sample sizes. Of the associated analytes, 10 to 468 proteins and 10 to 150 metabolites (Tables S4 and S5), depending on the genotype, were associated in the same direction in the replication dataset and passed multiple testing correction (FDR < 0.05) in the meta‐analysis (Tables S4 and S5).

In total, we identified 468 protein aptamers (185 up and 283 down) and 146 metabolites (50 up and 96 down) associated with AD risk in the *APOE*_33 genotype, 73 proteins (41 up and 32 down) and 73 metabolites (40 up and 33 down) for *APOE*_34, and 127 proteins (67 up and 60 down) and 92 metabolites (51 up and 41 down) for *APOE*_4X (Figure 1b, Tables S4 and S5). A heatmap of the average abundance of these proteins across APOE genotypes revealed distinct genotype‐dependent expression patterns, including both shared and APOE‐specific signals (Figure 1c). Among these proteomic biomarkers, Acetylcholinesterase (ACHE) was the only commonly up‐regulated protein across the four *APOE* genotypes (*APOE*_2X, *APOE*_33, *APOE*_34, *APOE*_4X). Fewer than 30 analytes were associated with AD vs. control for the *APOE*_2X, *APOE_*24 and *APOE*_44 genotypes, and therefore we focused on *APOE*_33, *APOE*_34, and *APOE*_4X genotypes for further analyses. Proteins associated with *APOE* genotype and cell‐type enrichment analyses (astrocytes, microglia, endothelial cells, neurons and oligodendrocytes) are presented in Figure S3 and S4.

To evaluate whether the choice of statistical framework affected the identification of AD‐associated proteins, we performed a sensitivity analysis using linear regression and compared the results with those obtained from the primary logistic regression model. Both analyses were applied to the same discovery, replication, and meta‐analysis pipelines within *APOE_33* and *APOE_34*. The two modeling approaches showed highly concordant results across all proteins (Figure S5, Tables S6 and S7). For *APOE_33*, 468 proteins were identified as differentially expressed in the logistic regression meta‐analysis, of which 461 (98.5%) were also significant in the linear regression model and exhibited identical effect directions. The −log_10_(P) values were extremely consistent between models (R^2^ = 0.997). Similarly, for *APOE_34*, all 73 protein associations identified by logistic regression were reproduced by the linear regression analysis, with 100% concordant effect directions and strong agreement in statistical significance (R^2^ = 0.996). These results demonstrate that the proteomic associations identified in *APOE*‐stratified analyses are robust and are not sensitive to whether AD status or protein abundance is modeled as the outcome.

For the genotypes with low sample sizes, splitting the samples into discovery and replication datasets might miss proteins with modest effects. To analyze whether this was the case, we further performed joint analysis (combining all samples from discovery and replication) and compared the *p*‐values and effect sizes in *APOE_2x* and *APOE_44* genotypes in the joint vs the meta‐analyses (Figure S6). The comparison results showed that the effect size values are highly correlated in the two settings (all Pearson correlation coefficients > 0.99). We further compared the differentially expressed proteins and metabolites (Table S3) and found that the same set of proteins and metabolites was identified in the two settings, with the meta‐analysis identifying one more metabolite in *APOE_44*. The R^2^ of the –log10(p_values) of the proteins and metabolites in the two settings was all greater than 0.95, indicating highly similar results between meta‐analysis and joint analysis.

#### 2.2.1 Replication of the *APOE*‐stratified Analyses in Independent Datasets and in Orthogonal Platforms

To further replicate and validate our findings, we leveraged SomaLogic‐based proteomic data from the GNPC, Indiana ADRC (IADRC), Bio‐Hermes and Standford ADRC (Figure S7 and Tables S8–S10). Each dataset was analyzed independently using the same proteomic QC, normalization and statistical model, and results from each dataset were meta‐analyzed. For a detailed description of the results for each dataset, see Extended Results. Overall, a total of 452 AD cases and 2,323 controls with an *APOE*_33 genotype and 419 AD cases and 1,091 controls with an *APOE*_34 genotype were included. We found that 83% of the proteins associated with AD in the *APOE_33* genotype presented the same direction and showed a significant *p*‐value in the replication dataset. For the *APOE_34* analyses, 81% of the proteins showed consistent direction and a significant association. An additional meta‐analysis of the Knight‐ADRC and the external datasets showed that all proteins had a consistent direction with the original association and were also associated with AD risk in the respective *APOE* genotype group, supporting the reproducibility of the findings across homogeneous proteomic platforms.

We also sought to replicate our findings using Olink as an orthogonal platform, using the UKBB and the Olink data from the Stanford ADRC. The sample size for this comparison was substantially smaller, as there was only a total of 171 AD cases in the *APOE*_33 and 290 AD cases in the *APOE*_34. The number of controls was large (N > 10,000), mainly coming from the UKBB, but this included many younger individuals not evaluated in memory clinics. Regardless of these limitations, we found that 91 (61%) and 20 (65%) of the overlapping proteins had the same direction of effect between the Knight‐ADRC and the Olink dataset in *APOE_33* and *APOE_34*, respectively (Table S11, Figure S8). The slightly lower replication rate may reflect differences in platform technology, limited protein coverage, and the imbalance between AD and control samples.

### 2.3 *APOE*‐Stratified Analyses Identify Additional Proteins Not Captured in Previous Studies Using Non‐Stratified Approaches

To further determine whether our *APOE‐*stratified association analysis identifies additional or different proteins than those found in studies that do not take *APOE* into account, we compared our results with several recent studies.

First, we compared our results with those of a recent study that performed proteomic analyses (Somalogic 7K) in 1,270 cases and 2,096 controls (Heo et al.) [36]. These are the same samples included in this study, enabling a head‐to‐head comparison of the *APOE‐*stratified vs the non‐stratified analyses. Of the 468 proteins associated with AD in *APOE*_33, 103 (22%) were also associated with AD in the non‐*APOE*‐stratified analyses and 365 (78%) were not. Similarly, 70% (*n* = 51) of the proteins associated with AD in individuals with the *APOE*_34 genotypes were not found in the overall AD analysis (Tables S12–S14, Figure S9). These results indicate that our analyses identify proteins and pathways that otherwise would not be identified in other general analyses. Some of the proteins that were associated with AD in the *APOE*_33 and *APOE*_34 genotypes but not in the non‐*APOE*‐stratified analyses included C3 and APOD. Those unique to *APOE*_33 include C4, BIN1, PPP3CA, PPP3R1, ITGA2B, or UNC5D. BIN1, ITGA2B and UNC5D have been involved in AD or other neurodegenerative diseases, and we and others have linked PPP3CA and PPP3R1 with pTau levels and rates of progression in previous studies [37]. Additionally, of the proteins identified in *APOE_33*, only 8 (TEC, SERPINF2, ACHE, NTN1, PDE5A, TFRC, SPON1, TIMP4) were reported as *APOE*‐independent and one (CTF1) as *APOE*‐dependent by Frick et al. [33], (Tables S12–S15, see Extended Results for additional analysis). None of the *APOE*_34‐specific proteins were reported by Frick et al. Therefore, 98% of proteins associated with AD, either in the *APOE*_33 or *APOE*_34 genotypes, were not reported as *APOE*‐independent in a study design using conditional analyses.

On the other hand, some of the previously identified AD‐associated proteins are also not captured by this study design. Heo et al. [36]., identified 456 aptamers associated with AD. Of those, 181 were found to be associated with AD in the *APOE*_33 group and 35 in the *APOE*_34 group. A total of 263 aptamers (59%) were not found associated with AD in either *APOE*_33 or *APOE*_34 (Tables S12–S14). A large proportion of those proteins not identified in the stratified analyses (SPC25, SOC25, LRRN1, NEFL, SMOC1, among others), are associated with *APOE* genotype in CSF and plasma according to recent proteogenomic studies [15, 38]. More importantly, those studies found that the association of *APOE* with those proteins was consistent in young healthy and AD biomarker‐free individuals, suggesting that those proteins may be associated with *APOE* and not with Alzheimer's disease. These findings further strengthen the overall design of this study, which is focused on identifying proteins associated with AD, but not driven by *APOE*, and that by performing analyses without stratifying by *APOE*, some of the identified proteins may be associated with AD only as a result of APOE's effect, not as a result of relevance to AD biology.

### 2.4 Common and Divergent Pathways Associated with AD Depending on the *APOE*‐Genotype

**FIGURE 2:**
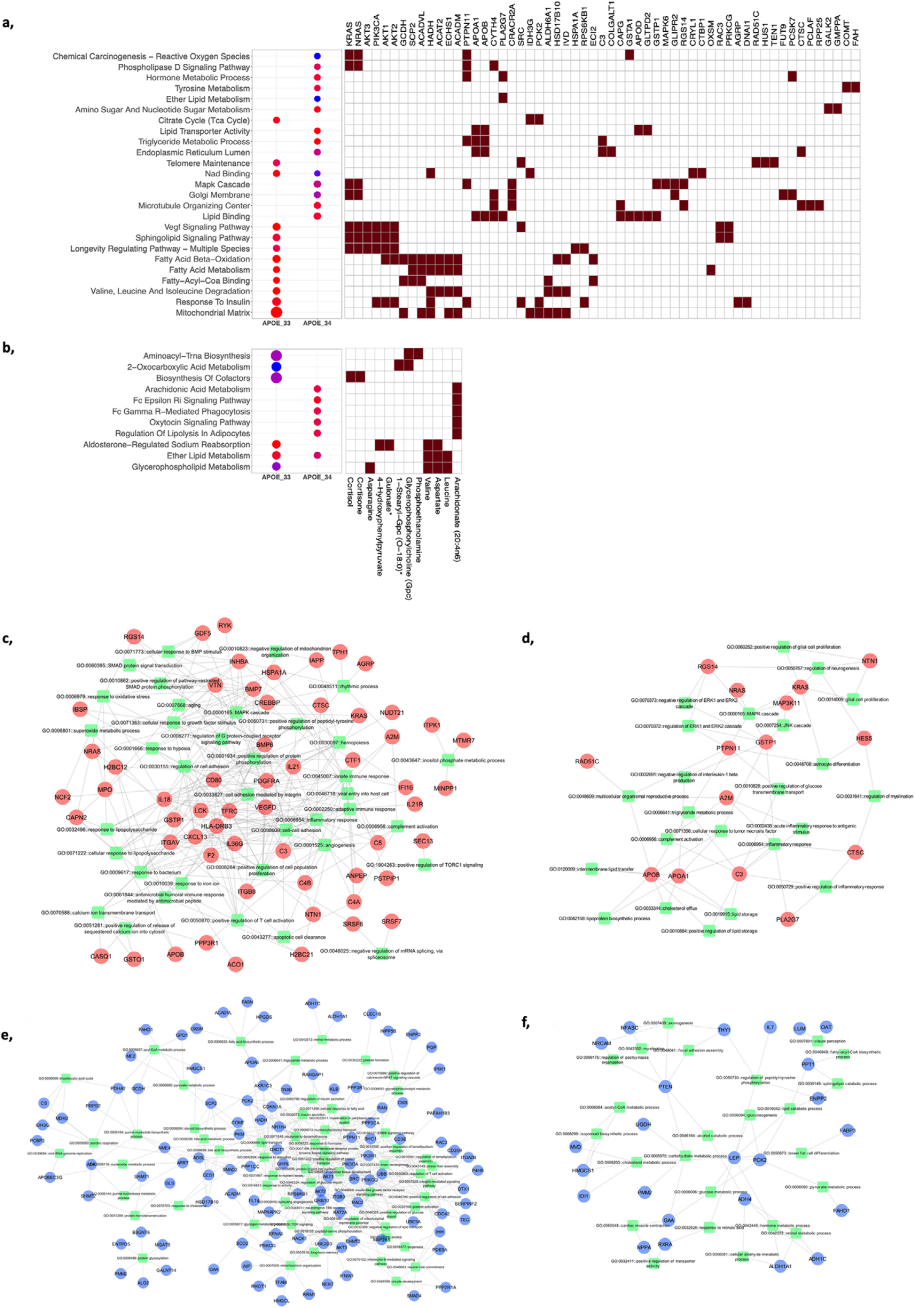
*APOE*‐stratified pathway and gene ontology networks in AD. (a,b) Dot‐Plots and targets associated with the enriched KEGG signaling pathways and GO terms using DEPs in *APOE*_33 (a) and in *APOE*_34 (b), respectively. (c–f) Enriched gene ontology (GO) terms in *APOE*_33 and *APOE*_34 genotypes. The association network of up‐regulated proteins and selected enriched GO terms of *APOE*_33 (c) and *APOE*_34 (d). The association network of down‐regulated proteins and enriched GO terms of *APOE*_33 (e) and *APOE*_34 (f). Blue and red circle nodes represent down‐ and up‐regulated proteins, respectively. The green square nodes represent GO terms.

We first performed pathway analyses using the proteins and metabolites associated with AD status in the *APOE*_33 and *APOE*_34 groups (Figure 2, Figure S10 **)**. In *APOE*_33, the proteins associated with AD capture several mitochondria‐related pathways, including branched‐chain amino acid (BCAA) degradation (*p =* 1.0 × 10^−5^), Mitochondrial Matrix (*p =* 7.7 × 10^−9^), Fatty Acid Beta‐Oxidation (*p =* 1.6 × 10^−7^), Fatty‐Acyl‐CoA Binding (*p =* 7.8 × 10^−5^), NAD Binding (*p =* 3.5 × 10^−4^), Citrate Cycle (TCA Cycle; *p =* 2.2 × 10^−3^), and Response to Insulin (*p =* 2.2 × 10^−3^; Table S16). In *APOE*_34, Phospholipase D Signaling Pathway (*p =* 1.1 × 10^−2^), Lipid Metabolism (*p =* 3.8 × 10^−2^), Reactive Oxygen Species (ROS; *p =* 3.8 × 10^−2^), Endoplasmic Reticulum (ER) Lumen (*p =* 1.9 × 10^−2^), Golgi Membrane (*p =* 2.6 × 10^−2^), and MAPK Cascade (*p =* 1.8 × 10^−2^; Figure 2, Table S17) were significantly enriched. These ROS and ER signaling pathways suggest that cells are experiencing oxidative stress and increased inflammation.

Analyses of the metabolomic and lipidomic data led to consistent findings with those from the proteomic analysis. Many lipid‐related pathways are associated with AD status in *APOE*_33 and *APOE*_34, including Ether Lipid Metabolism (*APOE*_33: *p =* 2.0 × 10^−2^; *APOE*_34: *p =* 4.5 × 10^−2^ (*APOE*_34) and Glycerophospholipid Metabolism in *APOE*_33 (*p =* 8.4 × 10^−2^; Figure 2b). *APOE*_33 specific pathways also implicate Aminoacyl‐tRNA Biosynthesis (*p =* 7.5 × 10^−2^) and 2‐Oxocarboxylic Acid Metabolism (*p =* 1.83 × 10^−2^) as the other top‐ranked metabolomic pathways. In *APOE_*34, immune signaling is the major associated pathway (*p =* 1.5 × 10^−2^, Figure 2b).

We also performed pathway analyses with those proteins that were associated with AD in both *APOE_33* and *APOE_34* (Figures S11 and S12, Table S18, and Extended Results). The identified pathways covered four main biological pathways involving the complement system, lipid and lysosomal metabolism, neuronal pathways, and basic metabolism (Extended Results). However, these analyses may underestimate the proteins and pathways associated with AD in the *APOE*_34 and *APOE*_33 groups due to different sample sizes in each analysis. Therefore, additional analyses are needed to determine truly *APOE*‐dependent and independent proteins and pathways associated with AD status.

#### 2.4.1 Identifying *APOE*‐Dependent and Independent Proteomic Signatures of AD

**FIGURE 3:**
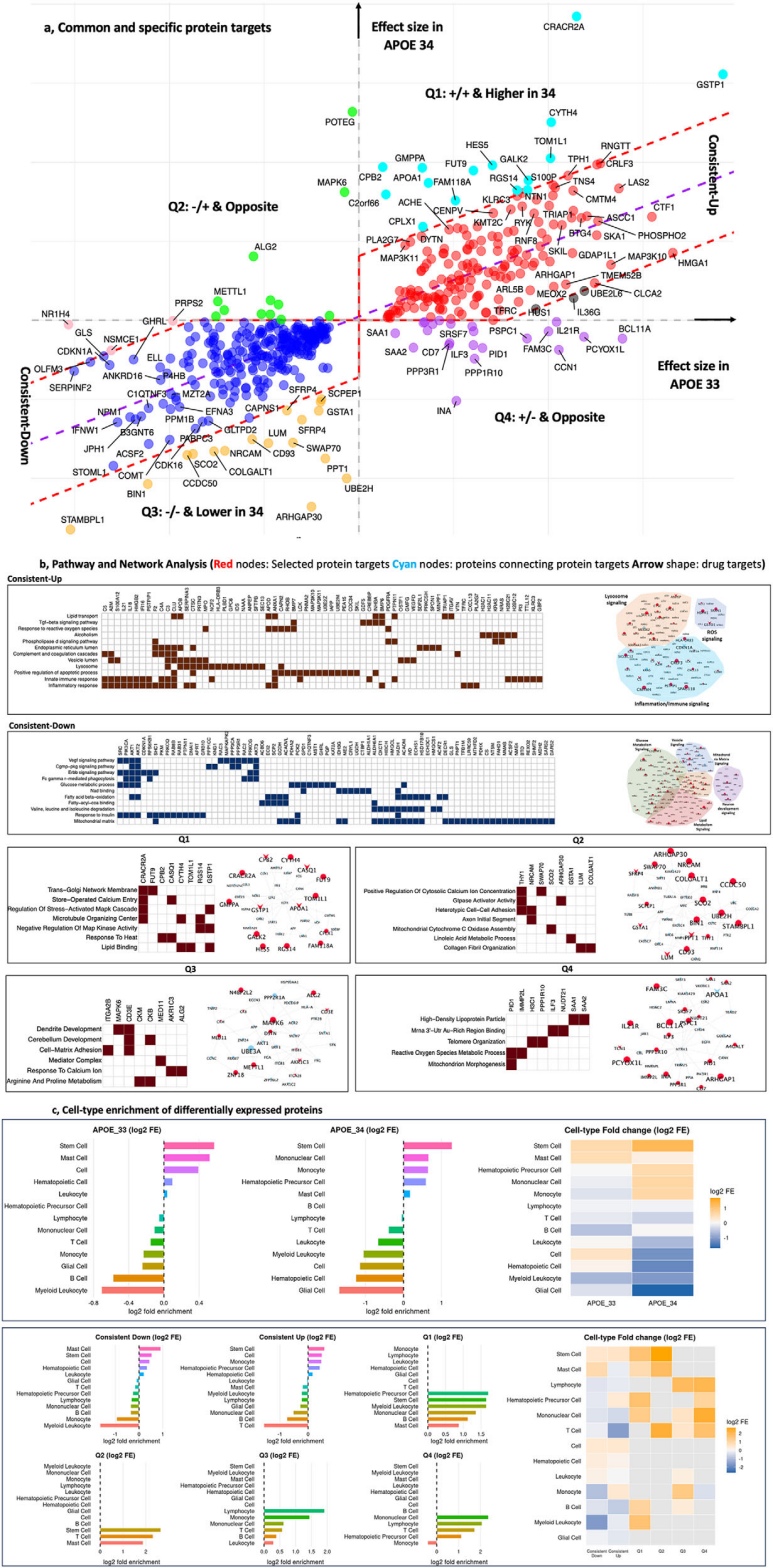
*APOE* stratified analyses identify converging and *APOE*‐specific proteins and pathways implicated on Alzheimer's disease pathogenesis. (a) Effect size correlation of the proteins associated with AD risk in *APOE*_33 and *APOE*_34. Most of the proteins have the similar effect size pattern (linearly correlated) in *APOE_33* and 34 genotypes. Specifically, there are in total 505 proteins identified from *APOE_*33 and *APOE*_34 genotypes. Among the 505 protein biomarkers, 433 (∼86%) proteins fall within the 3‐sigma range (±1.5‐sigma, 95% CI, indicated by the red dashed lines) of the central line (the purple dashed line based on linear regression of effect‐size points) in the first quadrant (consistent up) and the third quadrant (consistent down), and 72 showed different or opposite effect size. There are 15, proteins in the quadrant 1 (Q1; higher effect size in 34), 17 in Q3, 15 in Q2 (opposite effect size) and 25 in Q4 (opposite effect size). (b) Pathway and network analyses for the common up and downregulated proteins, as well as those in each quadrant. Red nodes: Selected protein targets Cyan nodes: proteins connecting protein targets Arrow shape: drug targets). Pathway analysis of the common proteins showed that mitochondrial and lipid metabolic processes are the major damaged signaling; and neuroinflammation, proteins in endoplasmic reticulum, lysosome and cellular vesicles are activated across *APOE* genotypes. The neuroinflammation is the consequent events of the damaged mitochondria and lipid accumulation. (c) Cell‐type enrichment of differentially expressed proteins. Log_2_ fold enrichment of DEPs across blood‐specific cell types in *APOE*_33 and *APOE*_34 groups and consistently downregulated and upregulated proteins, as well as Q1‐Q4 DEPs. The bar plots display enrichment for each cell type, and the heatmap summarizes the relative enrichment patterns.

To identify proteins associated with AD that are common between *APOE*_34 and *APOE*_33 or are *APOE‐*genotype specific, we compared the effect sizes of proteins associated with AD between those *APOE‐*genotypes. We defined proteins with similar effect size as those that fell within the 95% CI of the linear regression. These analyses revealed that 86% of proteins (486 aptamers: 270 down and 163 up‐regulated aptamers) showed similar effect sizes across these two *APOE* genotypes (Figure 1c, Figure 3a). We also found three proteins that have stronger positive effect sizes in *APOE*_33s (quadrant 1‐bottom) and 15 proteins with stronger negative effect sizes in *APOE*_34s (quadrant 1‐top). In addition, we also found proteins with opposite effect sizes across *APOE* genotypes. Specifically, there were 25 proteins with positive effect sizes in *APOE*_33 and negative effect sizes in *APOE*_34 (quadrant 4) and 15 proteins with the opposite trend (quadrant 2; Table S13).

#### 2.4.2 AD‐Associated Proteins in Common in *APOE*_33 and *APOE*_34 Individuals Are Enriched in Mitochondria and Lipid Dysfunction

Then, we performed pathway analysis for proteins that showed similar effect sizes between *APOE*_33 and *APOE*_34, consisting of 157 up‐regulated proteins and 263 down‐regulated proteins (Figure 3a, Table S19). The most significant pathway for the common down‐regulated proteins between *APOE*_33 and *APOE*_34 was the Mitochondrial matrix (GO:0005759, *p =* 1.49 × 10^−13^), which was driven by proteins such as FAHD1, PCK2, and ACSF2, all of which play basic functions in the mitochondria. Several pathways related to lipids and fatty‐acid metabolism (fatty acid beta‐oxidation; GO:0006635, *p =* 1.8 × 10^−10^; Fatty‐acyl‐CoA binding (GO:0000062, *p =* 4.0 × 10^−6^), were also among the top pathways. Several proteins from the AKT family are part of these lipid pathways as well as insulin‐related pathways (GO:0032868, *p =* 1.1 × 10^−4^), and Glucose metabolic process (GO:0006006, *p =* 3.4 × 10^−4^; GRB10, RPS6KB1, AKT1 among others). It is important to note that most of these pathways occur within the mitochondria (Figure 3b; Table S19). Network analyses were replicated using the omicsIntegrator2 [39] (See Extended Results and Figure S13) Cell‐type enrichment analysis based on peripheral blood reference profiles showed that the consistently regulated proteins were enriched in immune and hematopoietic cell populations. Specifically, the consistently downregulated proteins were enriched in mast and stem cells and depleted in myeloid cells and monocytes, whereas the consistently upregulated proteins showed depletion of T and B cells (Figure 3c). For completeness, we also evaluated enrichment patterns using brain cell‐type reference datasets. These analyses indicated that a subset of AD‐associated proteins was enriched in oligodendrocytes and neurons and depleted in microglia (Figure S14), suggesting potential relevance to brain‐resident cell populations, although these patterns likely reflect indirect or downstream associations rather than direct cellular sources of the plasma proteins.

The common up‐regulated proteins showed an enrichment in inflammatory and immune response pathways (Figure S3b). One of the most significant pathways, the complement and coagulation cascades (*p =* 3.5 × 10^−3^), includes known proteins identified by genetics such as CLU and other known AD‐related proteins such as A2M. C3, C4A and C5 are also part of this pathway. Negative regulation of the mitochondria organization (*p =* 8.6 × 10^−4^) was also significant and includes CLU as well as HSPA1A and IAPP. IAPP is one of multiple identified proteins associated with amyloid metabolism. In fact, the amyloid precursor processing pathway (*p =* 7.5 × 10^−3^; ACHE, SPON1 and FKBP1A) was also enriched in proteins found to be upregulated in *APOE_33* and *APOE_34* AD cases. Many pathways related to the immune system (innate immune response; *p =* 1.5 × 10^−2^; or Inflammatory response; *p =* 8.3 × 10^−3^; CTSC, APOD, SERPINA3; Figure 3b Table S16) were also significantly enriched in this group. This neuroinflammatory process might be connected with other mechanisms, including the activation of Response to Reactive Oxygen Species (ROS; GO:0000302, *p =* 2.7 × 10^−2^; APOD, PDGFRA, among others) which is also linked to mitochondrial dysfunction and damage. The ROS can also affect proteins in the ER lumen, another significantly enriched pathway (GO:0005788, *p =* 1.8 × 10^−2^; F2, CTSC, APOB among others). The activation of the lysosome (GO:0005764, *p =* 3.9 × 10^−3^; SERPINA3, C3, CAPN2 among others) could also be a consequence of digesting or breaking down the accumulated lipids or damaged proteins (Table S19).

Pooling the evidence of the pathways identified from the commonly up and downregulated proteins between *APOE*_33 and *APOE*_34, AD is associated with several changes involving damaged mitochondria and lipid accumulation that are reflected in the plasma. Neuroinflammation, ROS, ER and lysosome activation may be downstream of the damaged mitochondria and lipid accumulation.

#### 2.4.3 *APOE*‐Specific Proteins Capture Unique Process Implicated on Synaptic and Intracellular Trafficking

Although 86% of the proteins showed similar effect sizes between *APOE*_33 and *APOE*_34 genotypes, there were 15 proteins that showed higher positive effect sizes in *APOE*_34 (top‐region of Quadrant 1; **Q1‐top**) and three proteins (HUS1, IL36G, UBE2L6) that showed higher effect sizes in *APOE*_33 (**Q1‐bottom**). In addition, 17 proteins showed stronger negative effects in *APOE*_34 (**Q3‐bottom**), and three proteins (NR1H4, NSMCE1, PRPS2) showed a stronger decrease in protein levels in *APOE_*33 (**Q3‐top;** Figure 3a).

Of the fifteen proteins with higher effect sizes in *APOE*_34 *vs*. 33 (Figure 3a; Q1‐top), the protein with the largest effect difference was CPLX1, which plays a crucial role in synaptic vesicle exocytosis and neurotransmitter release. CASQ1 and CRACR2A, involved in calcium regulation, and CBP2 also showed higher effect sizes in *APOE_34* and is involved in the response to heat, which is related to energy production. These have not been reported to be associated with AD in previous studies [33, 40]. In addition, CASQ1 and GSTP1, another novel protein, regulate detoxification processes by catalyzing the conjugation of glutathione to various toxic substances, protecting cells from oxidative stress and damage. Other proteins in this group included TOM1L1 and CYTH4 (not identified in previous studies), implicated in vesicular trafficking, APOA1, involved in lipid metabolism, and GALK2 and FUT9 (novel proteins), involved in sugar metabolism. When examining peripheral blood cell‐type enrichment, these proteins were primarily enriched in hematopoietic and stem cell populations (Figure 3c). Consistent with these findings, brain cell‐type reference analyses showed that a subset of these APOE‐specific proteins were enriched in oligodendrocytes (e.g., CPB2) and astrocytes (e.g., HES5, FUT9) and depleted in neurons and microglia (Figure 3b, Figure S14).

In Q3‐bottom, 17 proteins that have stronger negative effect sizes in *APOE*_34 vs *APOE*_33 included known AD risk genes such as BIN1, which is involved in endocytosis and vesicular trafficking and is strongly genetically associated with AD risk [2]. The proteins included in this group are enriched in microglia (STAMBP1, ARHGAP30, SWAP70, CD93, SCO2, PPT1) and oligodendrocytes (SFRP4) and depleted of neuronal and astrocyte proteins (Figure S14), and are part of the cell‐cell adhesion (THY1, NRCAM), Cytosolic calcium (THY1, SWAP70) and collagen fibril organization (LUM, COLGALT1) pathways among others.

#### 2.4.4 Proteins With Opposite Effect Size Across *APOE* Genotypes

There were 34 proteins with opposite effect sizes between *APOE*_33 and *APOE*_34 (12 proteins with negative effect sizes in *APOE*_33 and positive in *APOE*_34 (**Q2**) and 22 proteins with the inverse pattern (**Q4**; Figure 3a)).

Proteins in Q2 are mainly expressed in astrocytes (AKR1C3, MED11) and in neurons (PRPS2, DSTN, MAPK6, NR1H4) and depleted in oligodendrocytes and microglia. Four proteins, METTL1, PRPS2, CKB|CKM and AKR1C3, are involved in general metabolic processes (protein and nucleotide synthesis; energy metabolism or steroid metabolism), while MAPK6, CD3E, and CKB are involved in dendrite development. AKR1C3 and ALG2 are related to the response to calcium ions (Figure 3b **, Q3;** Figure S14). Only one of the proteins in this group, MAPK6, has been associated with AD in previous proteomic studies, highlighting that this approach can identify proteins not captured by other study designs.

In Q4, 25 proteins were down‐regulated in A*POE*_34 and up‐regulated in *APOE*_33. The proteins in this group are mainly expressed in neurons (INA, PPP3R1, IMMP2L, FAM3C, NUDT21, PID1, BCL11A, PSPC1). Among these proteins, SAA1 and SAA2 are found in high density lipoprotein particles. PID1 and IMMP2L are associated with reactive oxygen species (ROS) metabolism. H3C1 and PPP1R10 are involved in telomere organization. PPP3R1 is the regulatory subunit of Calcineurin B, which has been implicated in tau phosphorylation and in the rate of AD progression [41]. (Figure 3b, Figure S14). None of the proteins in this group were identified in previous studies [15, 33].

### 2.5 *APOE*‐Stratified Metabolomic Analysis Indicate Lipid Dysregulation Beyond *APOE ε4*

**FIGURE 4:**
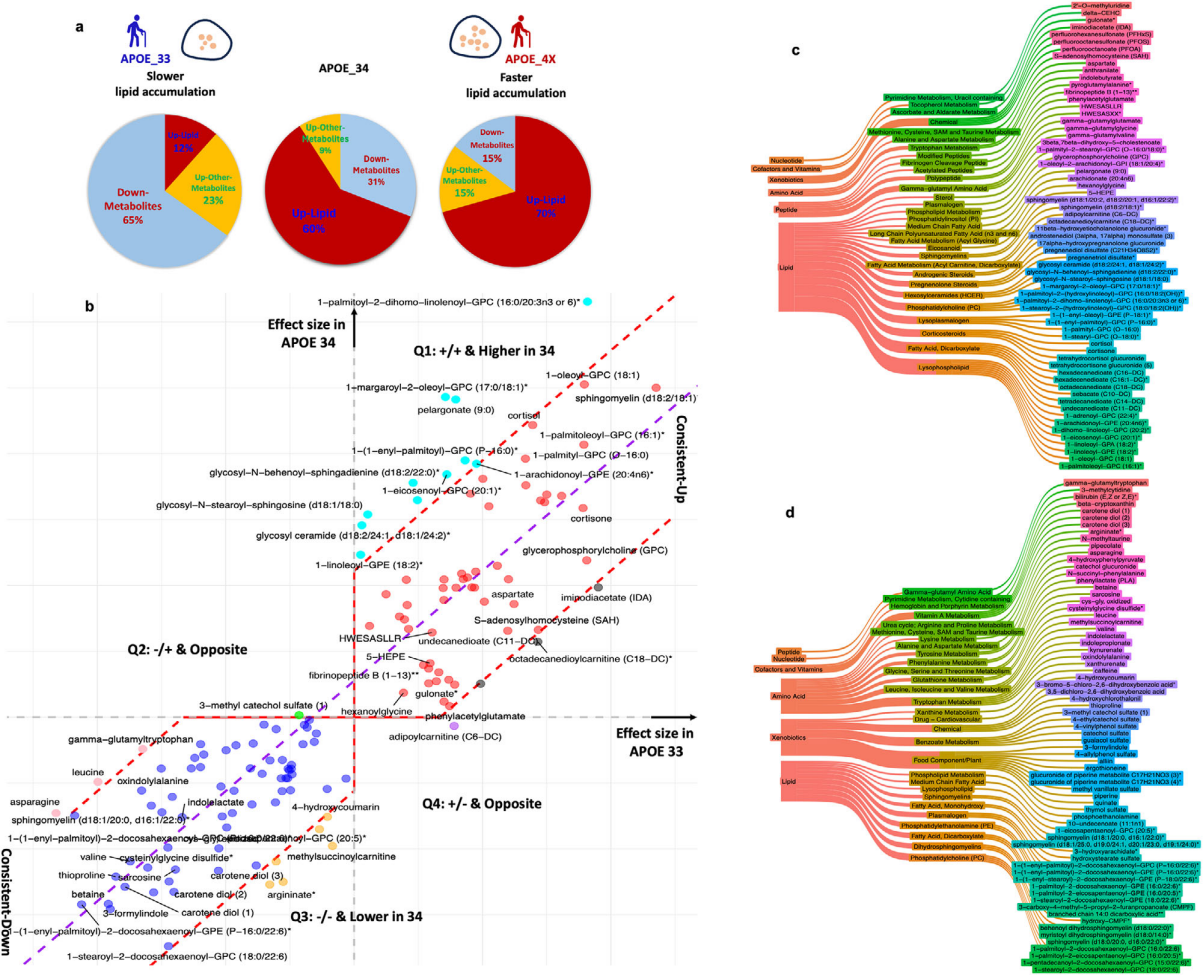
The *APOE ε4* allele is a factor exacerbating lipid accumulation. (a) Pie‐plots of up‐regulated lipid metabolites in *APOE_33* and *APOE_4x* AD samples. Specifically, only 12% are lipids in the differentially expressed metabolites in *APOE 33* genotype. Whereas the ratio increased to 60% and 70% in *APOE 34* and *APOE_44* genotypes, respectively. (b) Scattering plot of effect size of selected metabolomic biomarkers in *APOE_33* and *APOE_34* AD samples. Among the 137 metabolites, 112 (∼82%) metabolites fall within the 3‐sigma range (±1.5‐sigma, 95% CI, indicated by the red dashed lines) of the central line (the purple dashed line based on linear regression of effect‐size points) in the first quadrant (consistent up) and the third quadrant (consistent down), and 25 showed different or opposite effect size. (c,d) The Sankey plots of up‐ and down‐regulated lipids and their super‐ and sub‐pathways.

Our metabolomic analysis indicates that lipid dysregulation is a common process in AD and is regulated by additional mechanisms besides *APOE* (Figure 4a, Table S5). We identified 50 up and 96 down‐regulated metabolites in *APOE*_33 and 40 up and 33 down‐regulated metabolites in *APOE*_34. In the *APOE*_33 analysis only 12% of the associated metabolites were lipids, in contrast to 60% and 70% of the metabolites in the *APOE*_34 and *APOE*_*44* analyses. Moreover, this is mainly driven by lipids being up‐regulated (Figure 4b). These results indicate that there are additional *APOE*‐independent factors that exacerbate lipid accumulation.

After analyzing the effect sizes of the 137 metabolites associated with AD in *APOE*_33 and *APOE*_34, we found that 82% (*n* = 112) of the metabolites have similar effect sizes (Figure 4b). Specifically, there were 50 consistently‐up and 62 consistently‐down metabolites between the genotypes. At the same time, there were 11 metabolites with different effect sizes in Q1, 6 in Q3, one in Q2 (3‐methyl catechol sulfate) and one in Q4 (phenylacetylglutamate), respectively (Figure 4b, Table S20).

Among the 50 consistent‐up metabolites, 33 (66%) were lipids, 7 (14%) peptides, 4 (8%) amino acids, 3 (6%) xenobiotics, 2 (4%) vitamins. However, among the 33 lipids, 24 (73%) have higher effect sizes in *APOE*_34 carriers than in *APOE*_33 (Table S20), supporting the previous findings and the hypothesis that in *APOE*_34 individuals there are additional factors that exacerbate lipid accumulation (Figure 4b). The lysophospholipids (LPLs) are the major up‐regulated lipids in both *APOE*_33 and *APOE*_34 followed by fatty acids (Figure 4c, Table S20). LPLs are a subclass of plasmalogens within the phospholipid family that participate in various cellular processes including cell proliferation, differentiation, and apoptosis [42]. Dysfunction of LPLs has been associated with accumulation of amyloid‐β (Aβ) and formation of neurofibrillary tangles (NFTs). In addition, three Gamma‐glutamyl Amino Acids, which are bioactive peptides involved in inflammation, oxidative stress, and glucose regulation [42], are consistently upregulated in *APOE*_33 and *APOE*_34. The three xenobiotics that were associated with AD risk in these two *APOE* genotypes were perfluorooctanesulfonate (PFOS), perfluorooctanoate (PFOA), and perfluorohexanesulfonate (PFHxS; Figure 4b, Table S20). These results suggest that lipid dysregulation is consistently associated with AD but is more marked in *APOE_34* compared with *APOE*_33. This hypothesis is also supported by the metabolites found in the upper portion of Q1 (more strongly up‐regulated in *APOE*_34 than *APOE*_33), all of which (*n* = 11) were lipids including Phospholipids, LPLs, and Hexosylceramides (HCER; Table S20).

Of the 62 consistent‐down metabolites, 20 (32%) are lipids, 19 (31%) are xenobiotics, 16 (26%) are amino acids, and 5 (8%) are vitamins (Figure 4d, Table S20). Some of the downregulated metabolites come from food and plants, including caffeine, alliin (from garlic), quinate (from blueberry and apple), piperine (from pepper), and ergothioneine (from mushrooms/beans/oats), which have anti‐oxidation, anti‐inflammation and de‐toxifying functions. These data suggest that external environmental factors may be implicated in AD or that AD individuals have different behaviors or diets (Figure 4d). Among the consistent‐down amino acids (Figure 4d, Tables S20 and S21) kynurenate, oxindolylalanine, indolepropionate and indolelactate are part of Tryptophan metabolism signaling, which is important for circadian rhythms and nicotinamide adenine dinucleotide (NAD^+^) for energy generation. NAD^+^ also plays important roles in neurological function, mood regulation, immune response, and cellular energy production. In addition, some amino acids involved in the Glutathione metabolism pathway, like cys‐gly, oxidized, and cysteinylglycine disulfide, are down‐regulated in both *APOE*_33 and *APOE*_34. These are primary antioxidants and detoxifying agents. Moreover, some amino acids are in the Leucine and Valine metabolism pathway (leucine, valine, and methylsuccinoylcarnitine), which support some of the proteomic analyses that also identified these pathways (Figure 2). Finally, a set of vitamin A/B metabolites are down‐regulated, such as beta‐cryptoxanthin and carotene diol.

## 3 Discussion

Over the past few decades, technological leaps have allowed researchers to delve deeper into the genetic underpinnings of AD, yielding comprehensive genomic profiles that offer insights into potential risk factors, molecular pathways, and genetic mutations associated with the disease. Despite the considerable advancements in genomics and the generation of vast datasets related to AD, it is still unclear how the AD‐associated genes interact in specific pathways leading to disease. In addition to the genomic data, large‐scale proteomic profiles are being generated using mass spectrometry‐based and affinity‐based proteomics to measure the levels of thousands of proteins in complex biological samples, which can characterize and uncover dysfunctional signaling pathways or potential novel biomarkers [24, 27, 32, 33]. These studies also indicate that there are large metabolomic changes in AD [31, 43]. Therefore, large‐scale metabolomic and proteomic profiling of AD samples is critical to systematically understand the pathogenesis of AD. While there are several AD plasma proteomic and metabolomic studies, most of them do not try to disentangle the effects driven by *APOE* from those that are independent of *APOE*, which is the strongest genetic AD risk factor.

To fully understand the biology of AD, it is instrumental to identify *APOE*‐dependent and independent pathways. In this study, we analyze, for the first time, the proteins and metabolites associated with AD status stratified by *APOE* genotype. Specifically, we found 468 proteins associated with AD in *APOE*_33 and 73 proteins in *APOE*_34. In order to determine the novelty of our findings, we compared our study with Heo et al. [36] a large‐scale plasma proteomic study without *APOE* stratification using the same samples, and Frick et al. [33], who used statistical approaches to try to disentangle *APOE*‐dependent and independent proteins associated with AD. Our results indicate that most of the proteins (between 64%–78%) identified in this study are not identified in non‐*APOE‐*stratified analyses. At the same time, around 59% of the proteins associated with AD in the non‐stratified analyses were not associated with AD in the *APOE*_33 or *APOE*_34 genotypes. This could be due to two potential reasons. First, these two study designs may be capturing different processes, with the non‐stratified analyses potentially capturing proteins associated with AD that are driven by *APOE*. This hypothesis is supported by the fact that many of the proteins identified only in the non‐stratified analyses showed strong associations with *APOE* genotype in a recent pQTL study [15]. Another potential reason for the findings in our stratified analyses is due to the lower sample size and decreased statistical power due to stratifying the samples by *APOE* genotype. Frick et al., also aimed to identify *APOE*‐dependent and independent AD proteins, but they applied a statistical framework using an interaction term between *APOE* and AD status instead of performing a stratified analysis [33]. This approach can miss some *APOE* independent signals as well as overcorrect for the effect of *APOE*. In fact, most of the proteins (98%) we identified as associated with AD in the *APOE*_33 or *APOE*_34 genotypes were not identified by Frick et al. Additionally, most of the *APOE*‐dependent proteins (62%) identified by Frick et al., were also found by Heo et al. in the non‐stratified analysis, but we were only able to replicate 25% of their *APOE*‐independent proteins.

We acknowledge that this study indicates that the AD proteomic signatures in the *APOE*_33 or *APOE*_34 genotypes are remarkably similar. In fact, 83% of the proteins identified in this study associated with AD are shared between *APOE_33* and *APOE_34*. At the same time, this means that 17% are unique to each *APOE* genotype. These proteins can be important for identifying and understanding novel disease mechanisms. More importantly, more than 70% of these proteins are novel, illustrating how this approach helps to disentangle AD biology. The 17% of proteins that were uniquely dysregulated in one genotype are enriched in synaptic and intracellular trafficking and include some proteins never reported to be associated with AD, including CPLX1, TOM1L1, and CYTH4, which are implicated in vesicular trafficking. In addition, proteins associated with AD that are unique to *APOE_33* or *APOE_34* include protein products from known AD genes, such as BIN1, ITGA2B, and PPP3R1, that have not been associated with AD at the protein level in previous studies, showcasing that this approach will not only identify novel proteins but will also capture biologically relevant proteins and pathways that other approaches miss.

One of the significant findings of this study is the identification of mitochondrial and lipid metabolic processes as major pathways disrupted in AD in an *APOE*‐independent manner. This observation extends previous research that has consistently implicated mitochondrial dysfunction [44] and lipid dysregulation in the pathophysiology of AD [45]. Mitochondria are critical for energy production and cellular metabolism, and their impairment can lead to increased oxidative stress and neuronal damage, which are hallmarks of AD. These pathways were enriched for proteins (FAHD1, PCK2, ACSF2) associated with AD in both *APOE*_33 and *APOE*_34 and not found in previous plasma proteomic studies, in which most of the proteins and pathways are associated with *APOE* [33, 40].

The lipid and glucose metabolism are dysregulated in AD, potentially leading to decreased energy to support the activity of neurons. Several proteins from the ATK family (ATK1, ATK2), involved in lipid metabolism, may represent good therapeutic targets. The glucose metabolism pathway includes proteins such as PPP3CA, which is also a potential target with several FDA‐approved compounds, is associated with disease progression [41], and is an *APOE_33* specific protein. Other proteins associated with AD in both *APOE*_33 and *APOE*_34 (GPD1, SRC, AKT1) are also part of this pathway, together with other proteins (PCK2, PKM) that are part of the glucagon pathway. These analyses suggest that in AD, lipids are not properly converted into energy and instead accumulate inside or outside cells, which can cause inflammatory and immune responses (see the enriched pathways of consistent‐up proteins). This is critical for neurons because they consume the most energy to maintain continuous activity, ion gradients, and synaptic transmission. The brain, which contains billions of neurons, consumes about 20% of the body's total energy, despite only accounting for about 2% of the body's weight. To maintain the high energy demand, neurons have the largest number of mitochondria besides cardiac and skeletal muscle cells. Therefore, we hypothesize that mitochondrial damage is one of the key factors in AD pathogenesis. Our study's comprehensive proteomic analysis underscores the extent of mitochondrial damage, reinforcing the idea that AD is fundamentally a metabolic disorder initiated by mitochondrial dysfunction. Finally, the activation of neuroinflammatory pathways (CTSC, APOD, SERPINA3) and the involvement of proteins in the endoplasmic reticulum (F2, CTSC, APOB), lysosomes (SERPINA3, C3, CAPN2), and cellular vesicles across different *APOE* genotypes further elucidate the multifaceted nature of AD. Neuroinflammation is a well‐recognized feature of AD [46], contributing to disease progression through the release of pro‐inflammatory cytokines and the activation of glial cells. Our findings suggest that these inflammatory processes are not confined to a specific *APOE* genotype but are a common denominator in AD pathology. This broad activation of cellular vesicle‐related proteins highlights the potential for widespread intracellular transport and signaling disruptions in AD.

Moreover, the metabolomic analysis also captures the same pathways as those identified by proteomics, including lipid, mitochondrial, and glucose dysregulation. We identified a pronounced upregulation of lipid accumulation, particularly in individuals with the *APOE* ε4/ε4 and ε3/ε4 genotypes compared to those with the ε3/ε3 genotype. Importantly, this increase is not driven by *APOE*, as we compared cases and controls within the same *APOE* genotype. This lipid dysregulation, especially for lysophospholipids (LPLs) and fatty acids, is a critical insight, as it suggests that there may be other *APOE‐*independent mechanisms and genes driving this dysregulation. Lipids play vital roles in cell membrane integrity, signaling, and energy storage, and their dysregulation can have profound effects on neuronal function and survival. We observed the phenotypes of down‐regulated fatty acid β‐oxidation in proteomics data and increased lipid accumulation using metabolomics data. However, it is not enough to conclude a causal relationship between lipid accumulation and impaired β‐oxidation or decreased clearance via lipoprotein‐mediated pathways. There are a few studies that have reported the correlation between lipids and *APOE* ε4 in AD, but their causal relationship remains unclear. For example, alterations in brain lipids and metabolites have been reported in AD, and these changes correlate with neuropathological hallmarks of the disease and measures of cognitive decline [47, 48, 49]. Additionally, elevated total cholesterol (TC) and low‐density lipoprotein (LDL) levels were found in *APOE* ε4 allele carriers compared with non‐carriers [50, 51]. However, our results indicate that higher levels of triglycerides (lipids) are associated with increased AD risk in individuals who do not carry *APOE4* alleles. Although these studies indicated the importance of lipids and *APOE* ε4 interactions, their causal molecular mechanisms remain unclear. Moreover, it is important to note that the analyses performed in this study are based on plasma, and may not reflect changes in the brain, so additional validation in brain may be needed.

There are several limitations to this study that should be considered. This study was performed in plasma, which may not capture all the relevant pathways implicated in AD. There are several brain proteomic studies relevant to AD [22, 27, 52]. Johnson et al., used mass spectrometry proteomics in 516 brain samples and identified modules associated with AD implicated in MAPK signaling, metabolism, and the matrisome. More importantly, the matrisome module was associated with the *APOE* ε4 allele but not with the rate of cognitive decline. However, this study did not perform *APOE‐*stratified analyses, so it is not possible to determine how dependent the rest of the modules and proteins associated with AD are on *APOE*. Comparing the results from Johnson et al., with the findings presented in this study is not straightforward due to the different platforms used in each study. Seifar et al., also generated mass spectrometry proteomics in almost 1000 samples, which should provide good statistical power. One of the strengths of the Seifar et al., study is that it includes samples from different ancestries, which identified significant race‐related differences in amyloid and tau protein levels. However, this study design also makes it more difficult to replicate this study, as the number of samples of a specific *APOE* genotype and ancestry is limited. For example, in Seifar et al., for *APOE_33*, which should be the largest *APOE* genotype, there were only 53 AD and 69 control of European ancestry. Additionally, we did not analyze if any proteins or metabolites were sex or age specific. Recent studies have identified proteomic signatures specific to males or females [53, 54, 55, 56]. Therefore, further analyses stratifying by sex and *APOE* will be needed to fully disentangle the different pathways implicated in AD. In this study, we replicated the identified proteins in multiple datasets and across several platforms. However, this replication effort is may be confounded by the heterogeneity in the recruitment strategies, diagnostic criteria, and proteomic platforms across datasets. These differences can lead to both false‐positive and false‐negative findings. To limit the heterogeneity and especially the false positives, we performed a discovery and replication approach within the primary Knight‐ADRC dataset, from which all recruitment, sample collection, proteomic data generation, and QC was performed using the same protocols. Another limitation is that in this study, we focused most of the analyses on comparing the proteomics AD signatures in individuals with *APOE_33* or *APOE_34* genotypes, but additional analyses comparing all the other genotypes (i.e., *APOE_22 or APOE_44)* are needed. Although we compared the proteins found in each genotype (Figure 2), additional in‐depth pathway and network analyses will lead to the identification of additional pathways implicated in AD. We focused on the *APOE_33* and *APOE_34* genotypes, as those are the largest groups that provide more robust and replicable findings. To study the additional genotypes, larger studies will be needed.

In summary, this study advances the understanding of plasma proteomic, metabolomic and pathway dysregulation associated with AD in an *APOE*‐independent manner. This approach identified many proteins that were not captured by other non‐*APOE*‐stratified studies, as well as some specific proteins and disease mechanisms that are exacerbated within specific *APOE* genotypes. Specifically, our findings point to increased lipid accumulation and metabolic dysfunction as reflected in plasma in AD, which ultimately lead to mitochondrial dysfunction and altered synaptic and intracellular trafficking in AD pathogenesis. These insights may be able to guide the development of novel interventions aimed at mitigating mitochondrial damage and lipid dysregulation, helping to understand the pathways implicated on AD independent of *APOE*.

## 4 Materials and Methodology

### 4.1 Cohorts

#### 4.1.1 The Knight‐Alzheimer Disease Research Center

The Knight‐Alzheimer Disease Research Center (Knight‐ADRC) at Washington University in St. Louis has been at the forefront of groundbreaking research on Alzheimer's Disease. Extensive tissue and longitudinal data have been generated to advance research on dementia and aging. Specifically, the Genetics and High Throughput Omics Core (GHTO) [57] has gathered over 26 000 biological samples from 6625 participants, including DNA, RNA, non‐fasted plasma, cerebrospinal fluid pellets, and peripheral blood mononuclear cells. GHTO has conducted in‐depth molecular profiling on a large number of brain (*n* = 2117), CSF (*n* = 2012), and blood/plasma (*n* = 8265) samples to discover new risk and protective variants, molecular biomarkers, and potential therapeutic targets, which are crucial for deepening our understanding of AD. The plasma data used in this study were from the GHTO dataset. Specifically, Supplementary Table S2 shows the numbers of samples in the control and AD groups for each *APOE* genotype. As seen, *APOE*_2x (22/23) and *APOE*_33 had the latest mean onset ages for AD, at 79.43 years and 79.15 years, respectively. On the other hand, *APOE*_24 and *APOE*_34 had onset ages of 78.45 and 77.22 years, respectively, which are 1 year and 2 years earlier than *APOE*_2x and *APOE*_33, respectively. Patients with *APOE*_44 genotype had an average onset time of 73.07 years, which was 6 years earlier than that of *APOE*_33 patients. This difference in onset age could be attributed to the role of the APOE protein and associated biomarkers and signaling pathways in the accumulation and clearance of beta‐amyloid. Samples in the APOE genotype specific control groups are, on average, about 5 years younger than the AD groups. The female and male samples are well balanced across all *APOE* genotypes.

### 4.2 GNPC

The GNPC (Global Neurodegeneration Proteomics Consortium) is an international effort aimed at identifying biomarkers that enhance the understanding and treatment of neurodegenerative diseases. By consolidating proteomic data from thousands of patient samples across global dementia cohorts, the GNPC has assembled an extensive, harmonized proteomics dataset. This dataset was developed using both cross‐sectional and longitudinal data collected via the SomaScan platform (SomaLogic, Boulder, CO). Through collaboration, over 40 000 samples from more than 23 international cohorts were gathered, resulting in approximately 300 million protein measurements. This unified dataset covers various neurodegenerative conditions, including Alzheimer's Disease (AD), Parkinson's Disease (PD), Frontotemporal Dementia (FTD), and amyotrophic lateral sclerosis (ALS).

This study analyzed 3034 participants, with a mean age of 73.7 years, 43% male. To define case and control groups, clinical diagnoses such as AD, FTD, PD, MCI‐SCI, or recruited control status were prioritized. In the absence of clinical diagnoses, CDR scores were used: CDR = 0 indicated controls, CDR = 0.5 denoted MCI, and CDR > 0.5 signified dementia (case group). Where CDR and clinical diagnosis were unavailable, MMSE scores were used: MMSE > 24 for Control, 19–24 for MCI, and <19 for dementia (case). To maintain consistency, controls were excluded if coexisting disease diagnoses were present, and Control status was removed if CDR > 0 or MMSE ≤ 24. Additionally, AD status was removed if CDR = 0. Of the participants, 2522 were assigned to the control group and 512 to the case group. Proteomic data from the SomaScan 7K platform were utilized for analysis.

### 4.3 IADRC

The Indiana Alzheimer's Disease Research Center (IADRC) cohort includes dementia participants and cognitively normal (CN) elderly controls enrolled through the Indiana Memory and Aging Study (IMAS). All participants provided informed consent in accordance with the Declaration of Helsinki, and the study protocol was approved by the appropriate Institutional Review Boards. Participants underwent neurological and cognitive assessments using the Uniform Data Set 3 (UDS‐3) and provided blood samples for biomarker analysis. Diagnoses of Alzheimer's disease (AD) dementia were made using established criteria [58]. Cognitively normal participants were defined as older adults without significant cognitive impairment. The IADRC cohort includes individuals from diverse racial backgrounds, including White, Black or African American, and Asian.

A total of 302 participants from the IADRC cohort with available proteomic and clinical data were included in the present study. The average age was 68.0 years for participants with AD dementia and 69.4 years for cognitively normal controls, with 50.60% and 35.10% male participants in each group, respectively. Among the included individuals, 77 were diagnosed with AD dementia and 225 were cognitively normal controls.

### 4.4 Bio‐Hermes

The Bio‐Hermes study is a multi‐site observational cohort launched between April 2021 and November 2022, designed to evaluate biomarkers and digital tools for early detection of Alzheimer's disease (AD). A total of 1001 participants were enrolled across 17 research sites in the United States, all of which had experience recruiting for AD clinical trials. Participants were aged between 60 and 85 years and classified into three cognitive categories: cognitively normal (CN), mild cognitive impairment (MCI), or mild AD. All participants were required to have brain amyloid positivity and underwent extensive phenotyping, including MMSE assessment (scores between 20–30), *APOE* genotyping, and proteomic profiling using the SomaScan 7k assay. Additional details on the study design are available in Mohs et al. [59].

A total of 660 participants from the Bio‐Hermes cohort with available proteomic and clinical data were included in the present study. The average age was 74.45 years for participants with AD and 70.33 years for cognitively normal controls, with 48.46% and 38.64% male participants in each group, respectively. Among the included individuals, 277 were diagnosed with AD and 383 were cognitively normal controls.

### 4.5 Stanford‐Alzheimer's Disease Research Center

The Stanford ADRC cohort includes participants from a longitudinal observational study funded by the National Institute on Aging (NIA), along with individuals from the Stanford Aging and Memory Study (SAMS), an ongoing study of healthy aging. The cohort includes individuals with clinical dementia and cognitively normal controls, with cognitive status determined via standardized assessments and clinical consensus. All procedures were IRB‐approved, and informed consent was obtained from all participants or their legal representatives.

A total of 339 participants from the Stanford cohort with available SOMAscan‐based proteomic and clinical data were included in the present study. The average age was 70.97 years for participants with AD and 70.60 years for cognitively normal controls, with 50.91% and 40.85% male participants in each group, respectively. Among the included individuals, 55 were diagnosed with AD and 284 were cognitively normal controls.

A total of 363 participants from the Stanford cohort with available Olink‐based proteomic and clinical data were included in the present study. The average age was 70.45 years for participants with AD and 69.71 years for cognitively normal controls, with 51.67% and 39.93% male participants in each group, respectively. Among the included individuals, 60 were diagnosed with AD and 303 were cognitively normal controls.

### 4.6 UK BioBank Cohort Data Analysis

In UKBB dataset, there are 149 AD vs 26 159 control samples in *APOE*_33; and 252 AD and 10 484 control samples in *APOE*_34 genotype, respectively. Then, the UK Biobank Olink plasma proteomics data were used to identify the differentially expressed proteins.

### 4.7 Plasma Proteomics and Metabolomics Data of AD and Control Plasma Samples

The large‐scale proteomics (6907 features) and metabolomics (1508 features) datasets were generated from a total of 3060 samples, comprising 1655 control and 1362 AD samples. There are two datasets: 2200 samples were collected from year 1995–1997 and from year 2007–2021; and 860 samples were collected from year 1998 – 2006.

Blood was drawn from the peripheral veins, and the plasma was separated through centrifugation and subsequently preserved at a temperature of −80^0^C. The SomaScan v4.1 7K platform, which employs a multiplexed, single‐stranded DNA aptamer‐based technology from SomaLogic (located in Boulder, CO), was utilized to measure the proteomic data present in the plasma. Three distinct dilution sets (1:5, 1:200, and 1:20 000) were applied to plasma samples. To measure the protein concentration of 7584 modified aptamers, a relative fluorescent unit (RFU) readout was used, rather than physical units. To address technical variation resulting from pipetting errors, sample variance, and the readout, initial data standardization was carried out at both the sample and aptamer levels. This normalization was accomplished through a variety of methods, including hybridization control normalization, median signal normalization, and inter‐plate calibration, with the use of control aptamers (positive and negative controls) and calibrator samples*(70)*. To correct for systematic variability during hybridization, hybridization control normalization was implemented on each plate. Median signal normalization was then carried out within each dilution group to eliminate any bias introduced by pipetting variation, variations in reagent concentrations, and differences in assay timing, among other factors. Finally, inter‐plate calibration, which involved the use of calibrator samples, was carried out separately for each aptamer to eliminate any plate‐related bias.

Using SomaData (v1.8.0) and Biobase (v2.42.0), the proteomics data were further analyzed to remove aptamer outliers and sample outliers by using the following six‐step criteria [15, 60]. First, aptamer outliers were those with over 15% of samples having a concentration below 2 standard deviations above the average RFU level of the dilution buffer. Second, aptamer outliers were those with over 0.5 of the maximum difference between the aptamer calibration factor and the median of the plate calibration factor. Third, aptamer outliers had a median coefficient of variation (CV) greater than 0.15. Fourth, aptamer outliers had over 15% of samples with log10‐transformed RFU levels falling outside of either end of 1.5‐fold of the Interquartile range (IQR). Fifth, sample outliers had more than 15% of aptamers falling outside of either end of 1.5‐fold of the IQR. Finally, aptamer outliers were shared by approximately 80% of sample outliers. Following quality control procedures, a total of 6093 aptamers were deemed suitable for conducting differential abundance analyses. Universal Protein Resource (UniProt) [61] identifiers and Entrez Gene symbols were assigned to all 7584 aptamers.

Metabolomics: Samples were prepared using the automated MicroLab STAR system from Hamilton Company to remove proteins. All samples were measured using Metabolon's untargeted Precision Metabolomics LC‐MS. The technology is composed of four methods, including acidic positive ion conditions optimized for hydrophilic compounds, acidic positive ion conditions optimized for more hydrophobic compounds, basic negative ion optimized conditions, and negative ionization. All methods used a Waters ACQUITY ultra‐performance liquid chromatography, and a Thermo Fisher Scientific Q‐Exactive high‐resolution/accurate mass spectrometer interfaced with a heated electrospray ionization‐II source and Orbitrap mass analyzer operated at 35 000 mass resolution. The scan range varied but covered 70–1000 m/z.

There are 1508 metabolites (after‐QC) in plasma Metabolon data, of which 474 belong to the lipid category, in which there are 53 sub‐pathways. To group them based on localization or cellular function, there are 188 cell membrane lipids & their products, 55 hormone & their products, 164 fatty acids & their products, 37 belonging to bile acid metabolism, as well as 30 others (8 sub‐pathways) that stand alone.

### 4.8 Plasma Proteomics and Metabolomics Data Normalization

Specifically, large‐scale proteomics (6907 features) and metabolomics (1508 features) datasets of 1655 control and 1362 AD samples (in total 3060 samples) were analyzed. There are two datasets: 2200 samples were collected from year 1995–1997 and from year 2007–2021; and 860 samples were collected from year 1998–2006. For dataset1 and dataset2, the proteomics data were normalized respectively as: $p_{n,k}^{m}=log10\left(p_{n,k}^{raw,m}\right)$, where $p_{n,k}^{m}$ denotes the normalized protein level of the *m*‐th protein probe from raw protein data, $p_{n,k}^{raw,m}$, in the *n*‐th sample in the *k*‐th dataset. Then the removeBatchEffect function in the limma R package was employed on the log10 normalized protein level data to remove the batch effects. Then the two normalized datasets were merged together. The same normalization analysis was employed for the metabolomics data.

### 4.9 Discovery and Replication in the Knight‐ADRC Samples

For each *APOE* genotype, the AD and control samples were randomly clustered into the discovery and replication datasets. The discovery dataset was used to identify the *APOE* genotype specific biomarkers. The replication dataset was used as an independent dataset to validate the identified biomarkers. Supplementary Table S2 shows the *APOE* genotype specific number of AD and control samples in the discovery and replication datasets.

### 4.10 Identification of Differentially Expressed Proteins (DEP) and Metabolites (DEM)

The *APOE* genotype specific differentially expressed proteins (DEPs) were identified by using the logistic regression (LR) model (adjusted by Age and Sex), followed by the meta‐analysis. The LR model is defined as:

$$\mathrm{Ph}e_{i,j,k}∼\mathrm{Ag}e_{i,j,k}+\mathrm{Se}x_{i,j,k}+p_{i,j,k}^{m}$$

where, $i\in \left\{APOE_{2x=22|23},\;APOE_{24},\;APOE_{33},\;APOE_{34},\;APOE_{44},\;APOE_{4x=34|44}\right\},\;j\in \left\{\mathrm{Discovery},\mathrm{Replication}\right\},\;\;k\in 1,\;\;2,\;\;\text{…},\;\;n_{j}$ represents the k‐th sample in dataset j, and *Phe*_*i*,*j*, *k*_ $\in \left\{AD,\;\;Control\right\}$, and $p_{i,j,k}^{m}$ is the normalized protein level of the m‐th protein. The same model was employed to identify the differentially expressed metabolites.

For the meta‐analysis, the inverse variance weighted (IVW) fixed effect model was used. Specifically, for each protein probe, the pooled effect size and pooled standard error were defined as:

$$\beta_{pooled}=\frac{\sum_{n=1}^{N}w_{n}\beta_{n}}{\sum_{n=1}^{N}w_{n}}\text{, and}\;se_{pooled}=\sqrt{\frac{1}{\sum_{n=1}^{N}w_{n}}}$$

where β_*n*_ and *var*(β_*n*_) denote the effect size and variance of the effect size in the *n*‐th dataset (in this study *n* = 1,2 (discovery and replication datasets). The $w_{n}=\frac{1}{var(\beta_{n})}$ indicates the inverse variance weights (IVM). Then the $\chi_{df=1}^{2}$ statistics, following $\chi^{2}$ distribution with degree of freedom = 1, was calculated as:

$$\chi_{df=1}^{2}=\frac{{\beta_{pooled}}^{2}}{s{e_{pooled}}^{2}}$$

Subsequently, the corresponding *p*‐value for each protein was calculated as:

$$p_{meta}=p\left(x\geq \chi_{df=1}^{2}\right)$$

For multiple testing correction, the false discovery rate (FDR) was calculated using the Benjamini‐Hochberg (BH) procedure on the *p_meta_* values. Finally, the proteins that satisfied the following criteria were identified as DEPs:

$$p_{discovery}\leq 0.05\;and\;p_{replication}\leq 0.05\;and\;FDR\leq 0.05$$

### 4.11 Analyses in the GNPC

A total of 7290 analytes were included for the differential abundance analysis of protein levels between the AD and Control groups. Logistic regression models were conducted on the GNPC dataset, using clinical diagnosis (AD vs. Control) as the outcome and including sex, age at blood draw, contributor, and individual protein levels as covariates. The GNPC dataset was standardized using cohort‐specific Z‐scores based on log10‐transformed data. The analysis focused on the *APOE*_33 and *APOE*_34 genotypes. Unlike previous studies, the GNPC data were not divided into Discovery and Replication datasets. The analysis results from GNPC were subsequently integrated with previously conducted meta‐analysis results. This combined analysis factored in sample size and *p*‐values from both sources for an overall meta‐analysis performed using the metafor R package. An FDR *p*‐value threshold of <0.05, following the Benjamini‐Hochberg (BH) procedure, was applied to correct for multiple testing and establish statistical significance.

### 4.12 Analyses in the UKBB

The *APOE* genotype specific differentially expressed proteins (DEPs) were identified by using the logistic regression (LR) model (adjusted by Age and Sex), followed by the meta‐analysis. The LR model is defined as:

$$\begin{array}{ccc} Phe_{i,j,k} & ∼ & Age_{i,j,k}+Sex_{i,j,k}+blom\;rank-based\;inverse\\ & & normal\;transformation\;of\;protein\;level\\ & & +\;Olink\;batch+UKB\;sampling\;center \end{array}$$

### 4.13 Sensitivity Analysis Using Linear Regression

The primary analyses modeled AD status using logistic regression:

$$\mathrm{Status}\left(\mathrm{AD}/\mathrm{CO}\right)∼\mathrm{Proteins}+\mathrm{Age}+\mathrm{Sex}$$

To evaluate robustness across commonly used modeling frameworks, an additional sensitivity analysis was performed using linear regression:

$$\mathrm{Proteins}∼\mathrm{Status}\left(\mathrm{AD}/\mathrm{CO}\right)+\mathrm{Age}+\mathrm{Sex}$$

Both logistic and linear regression analyses were applied to the *APOE*_33 and *APOE*_34 groups using the same samples, covariates, and discovery, replication, meta‐analysis procedures. Concordance between the two approaches was evaluated by comparing effect directions and *p*‐values across all proteins (Figure S5, Tables S6 and S7).

### 4.14 Sensitivity Analysis for Meta vs Join Analyses

We calculated the effect size and *p*‐values in the joint‐analysis (by pooling all samples from both discovery and replicate datasets) using the same logistic regression model for the analysis in discovery and replicate datasets. Then the FDR was calculated by applying the Benjamini‐Hochberg (BH) correction on the *p*‐values. Then we compared the effect size, *p*‐values and selected targets between meta‐analysis and joint‐analysis in the *APOE_2X* and *APOE_44* genotypes (Figure S6, Table S3).

### 4.15 KEGG Pathway Enrichment Analysis

Then the *p*‐value of each KEGG pathways was calculated using the Fisher's exact test, which compares the numbers of DEPs in a given KEGG signaling pathway and the number of DEPs in the remaining proteins that do not belong to the given KEGG signaling pathway.

### 4.16 Gene Ontology (GO) Enrichment Analysis

To conduct the GO enrichment analysis, the following two R packages: ‘GO.db’ (to obtain the GO term id and names using the ‘Term’ and ‘Ontology’ functions) and ‘org.Hs.eg.db’ (to extract the genes in each GO using the ‘get’ function) were employed. The *p*‐value of each GO was calculated using the Fisher's exact test, which compares the number of DEPs in a given GO and the number of DEPs in the remaining proteins that do not belong to the given GO. Then the *p*‐values are used to identify activated biological processes (BP) gene ontology (GO) terms. Specifically, the activated GO terms with genes in [10, 500] and *p*‐value ≤ 0.05 were identified.

### 4.17 Cell Type Specificity Analysis

Reference cell type expression profiles were downloaded from publicly available single‐cell RNA‐sequencing datasets from 10× Genomics. Gene expression matrices were processed to obtain average expression levels for each annotated cell type. For each gene, total expression across the 13 reference cell types was calculated by summing its mean expression values across all cell types. The proportional contribution of each cell type to this total was then determined. A gene was classified as cell‐type specific when the highest contributing cell type accounted for at least 1.5× greater expression than the second‐highest cell type. Proteins quantified by the SomaScan 7k platform were mapped to Entrez Gene symbols, and each protein was assigned to a cell type based on the specificity of its corresponding gene. Cell type specific gene counts were used to estimate the representation of each cell type in the DEP sets. Cell type enrichment analysis was performed to evaluate DEPs across *APOE_33, APOE_34*, consistent, and Q1–Q4. DEPs were mapped to reference signatures representing 13 major human cell types, including Stem Cell, Mast Cell, Cell, Hematopoietic Precursor Cell, Lymphocyte, Mononuclear Cell, T Cell, Monocyte, Glial Cell, B Cell, Erythrocyte, Hepatocyte and Myeloid Leukocyte. For each protein subset, the number of genes assigned as specific to each cell type was determined. Bar plots and heatmaps were generated to visualize the distribution of cell types (Figure 3c).

### 4.18 Quadrant Analysis of Proteomics Data of APOE_33 and APOE_34 Cohorts

The differentially expressed proteins (DEPs) in *APOE_33* and *APOE_34* sub‐cohorts were merged together. A linear regression analysis was used to identify the center line fitting the 2D effect size space of the DEPs, that is, effect size in *APOE*_33 cohort (x‐axis), and effect size in the *APOE_34* cohort (y‐axis). Then the distances of all the data points in the 2D effect size space to the center line were calculated, and the standard deviation (σ) of the distances was calculated. Then the commonly up and down regulated proteins in *APOE*_33 and *APOE*_34 were identified as the DEPs falling within the 3σ of the center line. Consequently, the remaining DEPs located outside the 3σ boundary of the center line were identified in each quadrant.

### 4.19 Signaling Network Inference Using Integrative Interactomes

The protein‐protein interaction (PPIs) in BioGrid [62] were employed as the background network to infer the potential signaling interaction networks among the DEPs. Specifically, let $G_{0}=\left\langle R_{0},\emptyset \right\rangle$ denote the initialized null signaling network starting from the given root nodes *R*_0_. The root nodes are the proteins with higher node degrees. Then the network grows from the root nodes iteratively as follows: $G_{t+1}=f\left(G_{t},G_{B},V_{K}\right)$, where $G_{t}$ and *G_B_* represent the current inferred signaling network and background signaling network, respectively. *V_K_* is a vector including *K* remaining candidate proteins in the DEPs. For any protein, $p_{k}\in node\left(G_{t}\right)$, the shortest paths from *p_k_* to all the candidate proteins in *V_k_*, are then calculated. Then the protein in *V_k_* that has the shortest path to one of the nodes of $G_{t}$ and the associated proteins on the shortest path, is selected and added to the network. The network growth process stops once all DEPs are added to the signaling network.

To analyze how robust the networks are, we also performed network analyses using an alternative approach. Specifically, we used the established network analysis omicsIntegrator2 [39]. The omicsIntegrator2 (OI2) was developed to integrate multi‐omics data, and map them onto signaling networks. Then the prize‐collecting Steiner forest (PCSF) [63] model was employed to identify subnetworks. We employed the PCSF algorithm to generate the signaling networks based on the selected pathway‐specific DEPs in consist‐up, consist‐down, and Q1, Q2, Q3, Q4. Both the OI2/PCSF and shortest path approaches have the same protein nodes as input, and identify the potential interactions among these nodes.

### 4.20 Identification of FDA Approved Drugs Inhibiting Genes on the Uncovered Signaling Network

The FDA approved drug and their target information was derived from drugbank [34] database. Specifically, the ‘Target Drug‐UniProt Links’ file of ‘Approved’ drugs (the FDA drugs) was downloaded from the ‘EXTERNAL LINKS’ tab on the ‘Downloads’ page of DrugBank website to identify the FDA drugs; and associated drug‐target information. Then the FDA approved drugs inhibiting the selected genes were identified as drug candidates that can potentially perturb the uncovered signaling network. In addition, using the consist‐up regulated proteins and the consist‐down regulated proteins as the biomarkers for drug repositioning using the clue.io API in the connectivity map (CMAP) database [35]. Specifically, the gene set enrichment analysis (GSEA) [64] algorithm was used to rank drugs that can potentially reverse abundance of these proteins. Then the top ranked drugs were selected and analyzed (Table S21).

For the druggable genome [65], we leveraged information based on the reported data that include 1427 tier‐1 druggable proteins (targets of approved drugs); 682 tier‐2 proteins (targets of bio‐active drug‐like small molecules; and 2370 tier‐3 proteins (with distant similarity to the targets of approved drugs).

## Funding

This work was supported by grants from the National Institutes of Health (R01AG044546 (CC), P01AG003991(CC, JCM), RF1AG053303 (CC), RF1AG058501 (CC), U01AG058922 (CC), RF1AG074007 (YJS), P30AG10161 (DAB), P30AG72975 (DAB), R01AG15819 (DAB), R01AG17917 (DAB), U01AG46152 (DAB), U01AG61356 (DAB)), 1R21AG078799‐01 (FL), R4R33AG078799‐02(FL), the Chan Zuckerberg Initiative (CZI), the Michael J. Fox Foundation (CC), the Alzheimer's Association Zenith Fellows Award (ZEN‐22‐848604, awarded to CC), and an Anonymous foundation.

The recruitment and clinical characterization of research participants at Washington University were supported by NIH P30AG066444 (JCM), P01AG03991(JCM), and P01AG026276(JCM).

The recruitment and clinical characterization of research participants at Washington University were supported by NIH P30AG066444 (JCM), P01AG03991(JCM), and P01AG026276(JCM).

This work was supported by access to equipment made possible by the Hope Center for Neurological Disorders, the Neurogenomics and Informatics Center (NGI: https://neurogenomics.wustl.edu/) and the Departments of Neurology and Psychiatry at Washington University School of Medicine.

## Conflicts of Interest

CC has received research support from GSK and EISAI. CC is a member of the scientific advisory board of Circular Genomics and owns stocks. CC is a member of the scientific advisory board of ADmit. There is an invention disclosure for the prediction models, including protein IDs, alternative proteins and weights, cut off and algorithms. DMH is as an inventor on a patent licensed by Washington University to C2N Diagnostics on the therapeutic use of anti‐tau antibodies. D.M.H. co‐founded and is on the scientific advisory board of C2N Diagnostics. D.M.H. is on the scientific advisory board of Denali, Genentech, and Cajal Neuroscience and consults for Pfizer, Roche, and Switch. The remaining authors declare no competing interests.

## Code Availability

All analyses were conducted in R (v4.4.0). Proteomic matrices were processed using SomaDataIO (v1.8.0) and Biobase (v2.64.0). Data manipulation and integration were performed using the dplyr (v1.1.4), plyr (v1.8.9), and tidyr (v1.3.1) packages. Differential abundance and odds ratio analyses were carried out using linear models (lm) from the stats package (v4.4.0). Meta‐analysis was implemented through a custom method using weighted Z‐scores (based on inverse‐normal transformation of *p*‐values) and weighted averages of effect sizes, with sample size as weights. Correlations and *p*‐values were calculated using cor.test() in R.

Pathway enrichment analyses were performed using the clusterProfiler (v4.12.6) and ReactomePA (v1.48.0) packages for Gene Ontology and Reactome pathway databases, respectively. Data visualization utilized ggplot2 (v3.5.1) and grid (v4.4.0). Protein‐protein interaction (PPI) networks and functional modules were derived using the BioGRID database and customized network‐based analysis scripts in R.

## Supporting information




**Supporting File**: advs74237‐sup‐0001‐SuppMat.pdf.

## Data Availability

The data that support the findings of this study are openly available in GNPC at https://www.neuroproteome.org/, reference number 1.
