# Supplementary material for: APOE‐stratified Proteomic and Metabolomic Analysis Reveals Mitochondrial Dysfunction Inflammation and Lipid Dysregulation in Alzheimer's Disease
Source: Adv Sci (Weinh). 2026 Feb 5;13(21):e13872. doi: 10.1002/advs.202513872 (PMC13073338; doi:10.1002/advs.202513872)

Alzheimer’s disease is a metabolomic disorder driven by mitochondrial damaging revealed by *APOE* allele-independent proteomic and metabolomic analysis

Supplementary figures

Figure S1: **Age distributions of control and AD in different *APOE* genotypes.**
a, Genotype-specific age distributions of control and AD samples. b, Age distributions in AD samples across different *APOE*-genotypes. c, Age distributions in control samples across different *APOE*-genotypes.

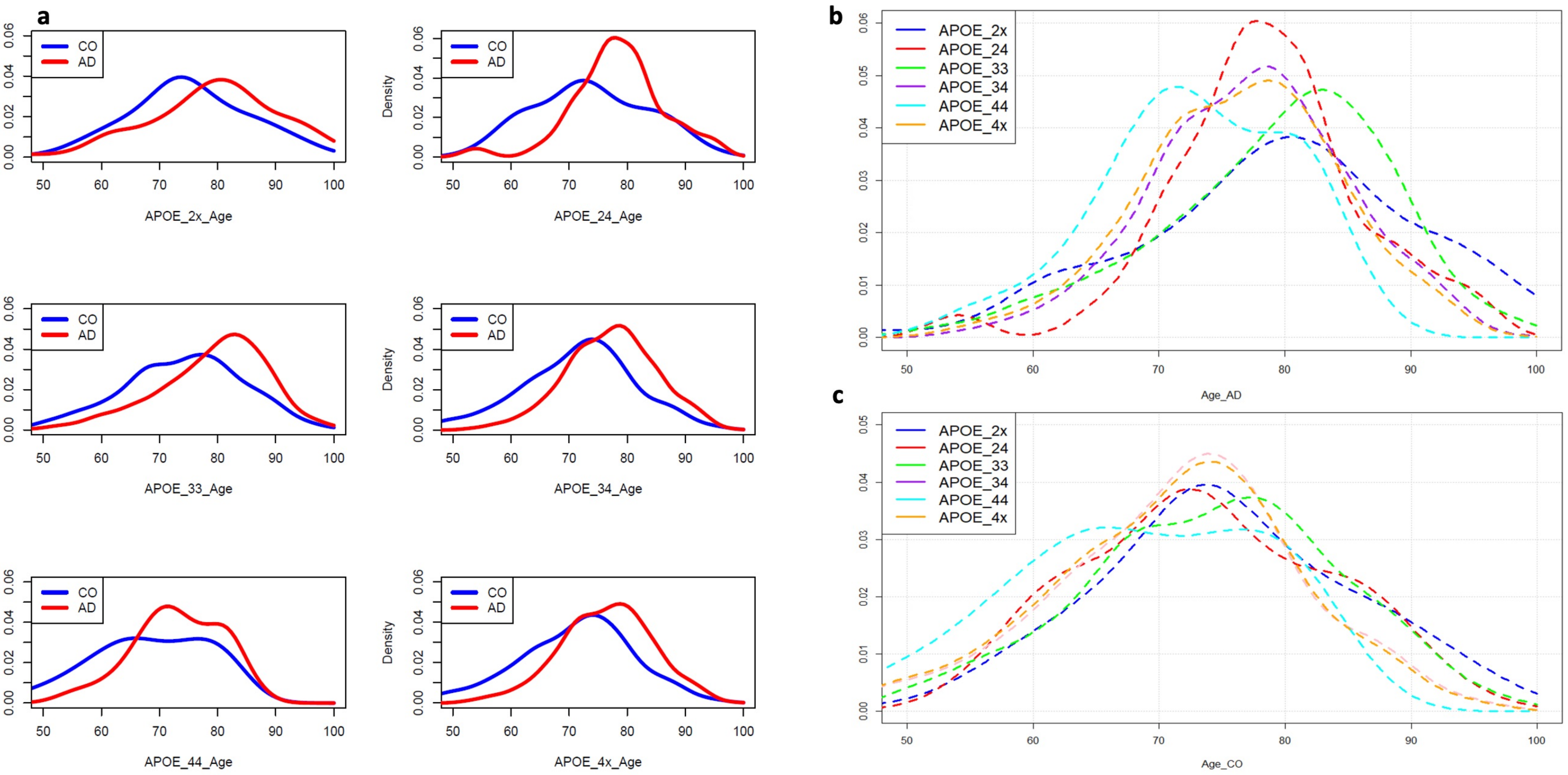

Figure S2: Volcano plots in different *APOE* genotypes.

a-f, Volcano plots (x-axis: pooled beta value; y-axis: q-value of meta-analysis) of *APOE*_2x (22/23) (a), *APOE*_24 (b), *APOE*_33 (c), *APOE*_34 (d), *APOE*_44(e), *APOE*_4x(f). Red dots indicate proteins or metabolites with q-value <= 0.05.

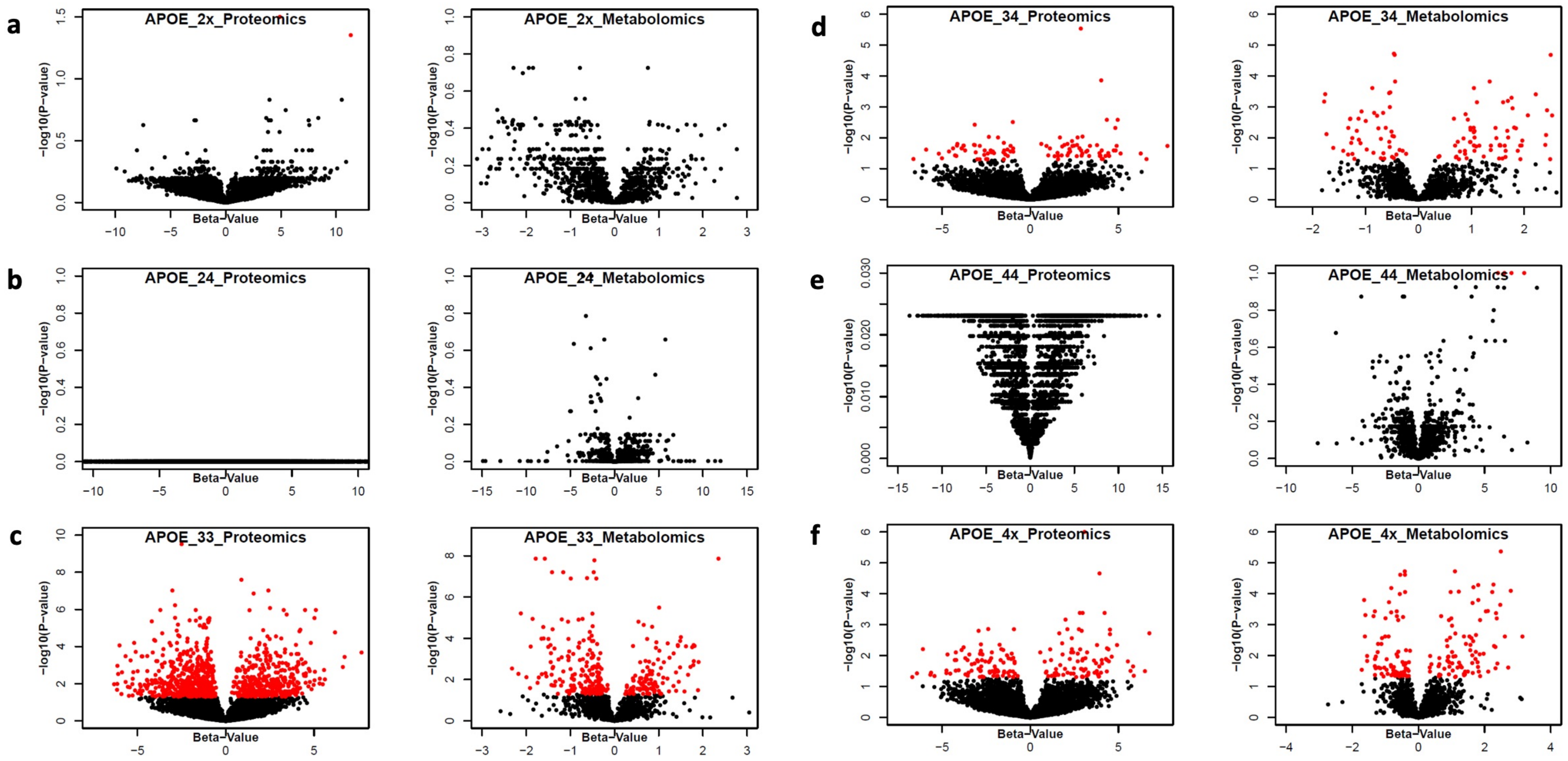

Figure S3: ***APOE*** **genotype-protein-Cell type association network.**

a,b, The up-(a) and down- (b) regulated proteins after the direction analysis in the discovery and replicate datasets in different *APOE* genotypes. There are 18 (13 up, 6 down) in *APOE*_2x, 7(5 up, 2 down) in *APOE*_34, 453 (177 up, 276 down) in *APOE*_33, 102 (54 up, 48 down), 8 (3 up, 5 down), 164 (79 up, 85 down) differentially expressed proteins respectively.

c.d, The up-(c) and down-(d)regulated proteins in different *APOE* genotypes and associated cell types. There are 30, 38, 14, 44, 10 up-regulated proteins; and 37, 36, 15, 66, 18 down-regulated proteins in astroctyes, microglia, endothelial, neurons and oligodendrocytes respectively.

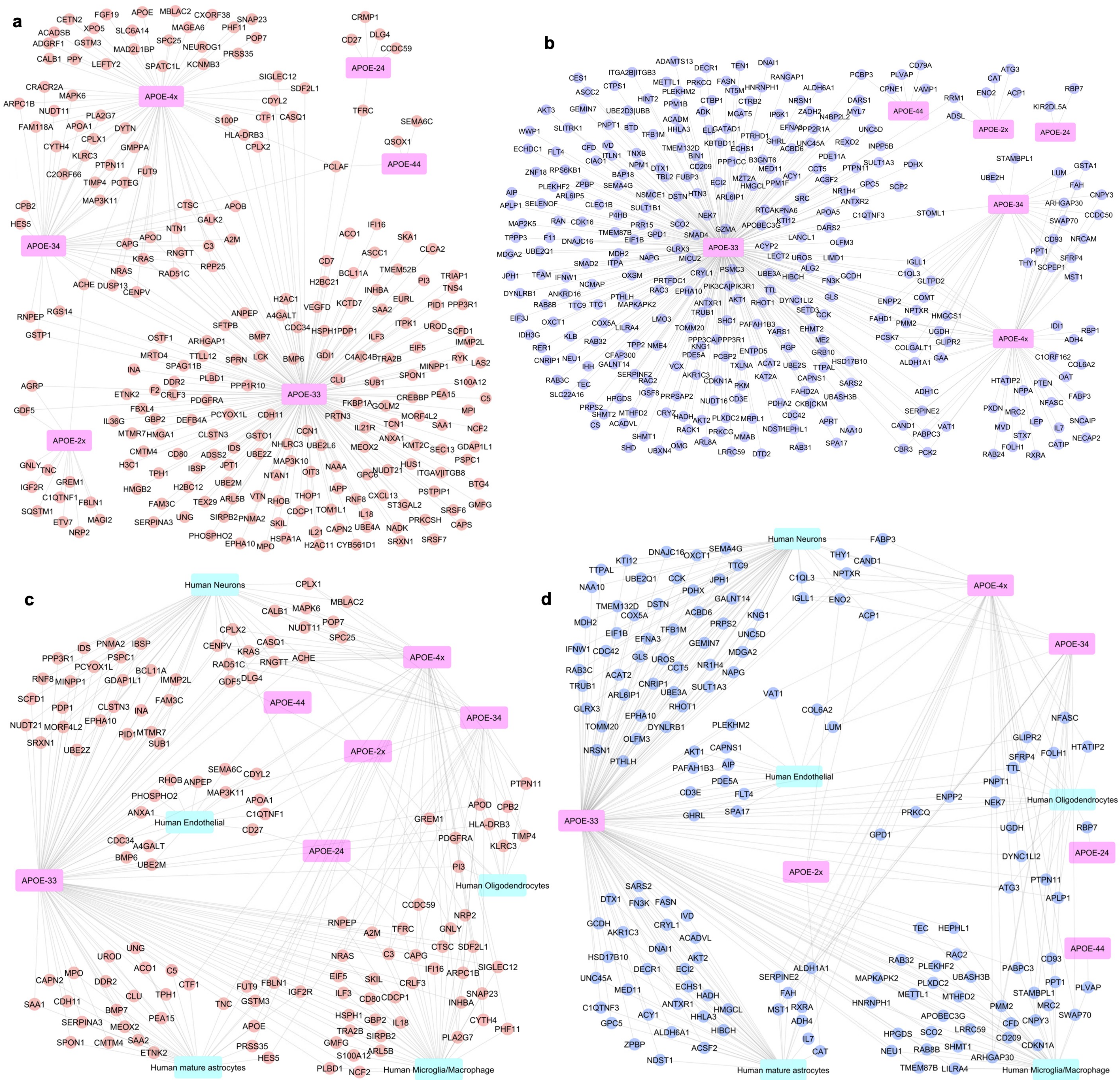

Figure S4: Enrichment analysis of cell types.

Enrichment analysis of five cell types (astroctyes, microglia, endothelial, neurons and oligodendrocytes) using the up (**a**) and down (**b**) regulated proteins after the direction analysis in discovery and replicated datasets for each genotype. There are 18 (13 up, 6 down) in *APOE*_2x, 7(5 up, 2 down) in *APOE*_34, 453 (177 up, 276 down) in *APOE*_33, 102 (54 up, 48 down), 8 (3 up, 5 down), 164 (79 up, 85 down) differentially expressed proteins respectively.

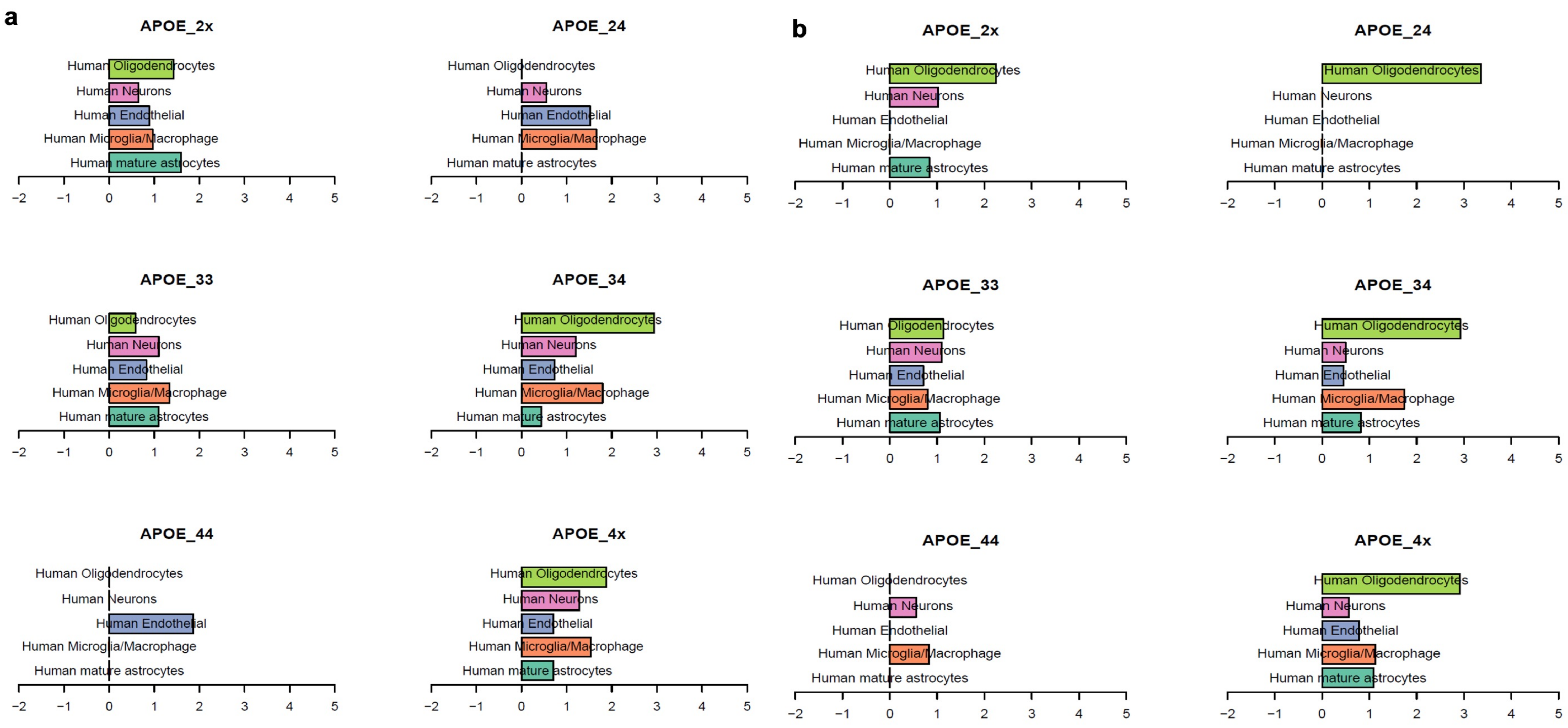

Figure S5. Effect size comparison of the logistic and linear regression.

(a) Scatterplots comparing -$\log_{10}$(p) values from logistic and linear regressions in *APOE*_33, in the discovery, replication and the meta-analyses. **(b)** Scatterplots comparing -$\log_{10}$(p) values from logistic and linear regressions in *APOE*_34.

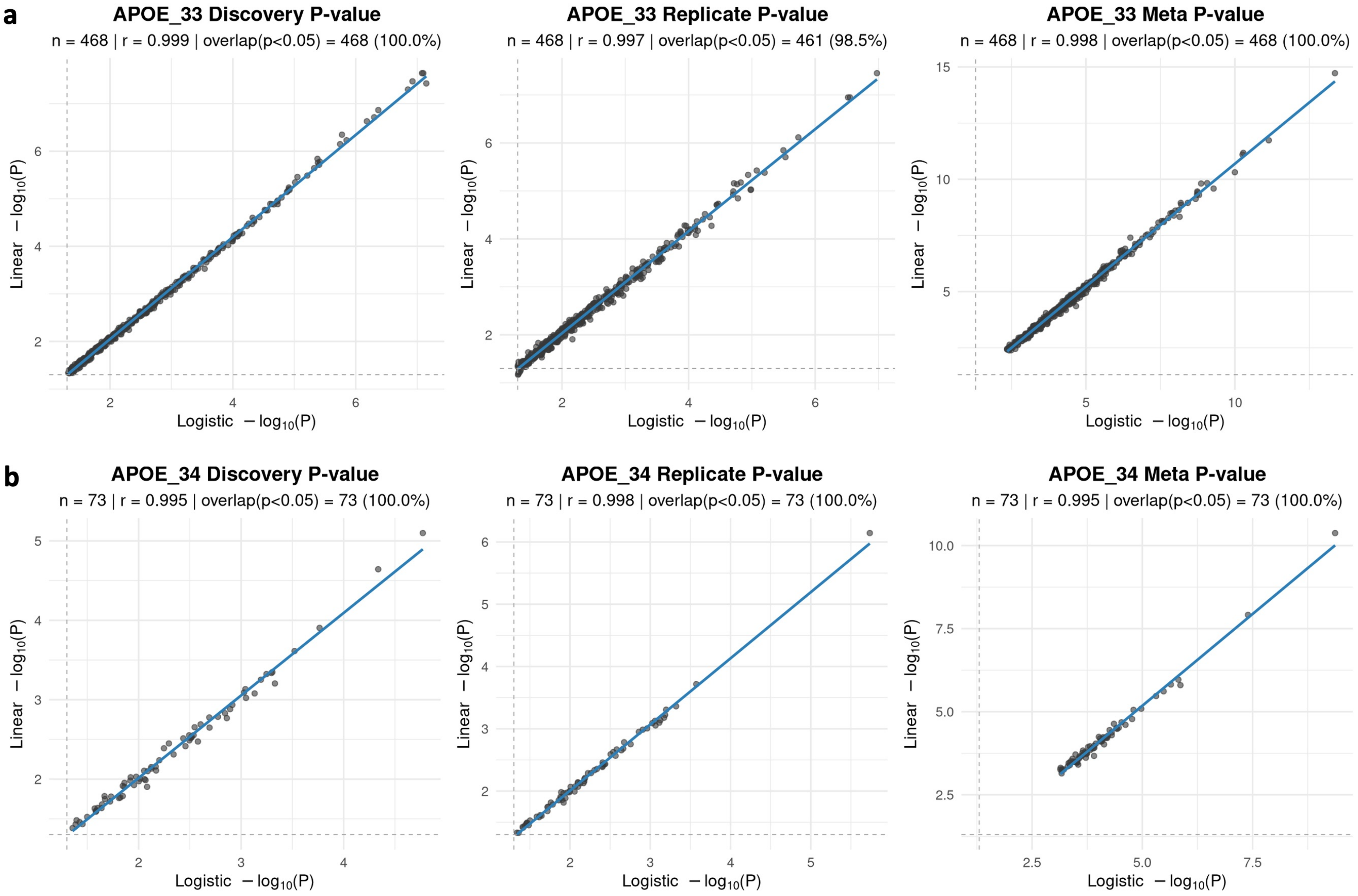

Figure S6: Effect size comparison between the meta-analysis vs the join analyses (pooling all samples). The scatter plots show the correlation of the effect size values of proteomics and metabolomics data in *APOE*_2X (a) and *APOE*_44 (b). All Pearson correlation coefficients are greater than 0.99.

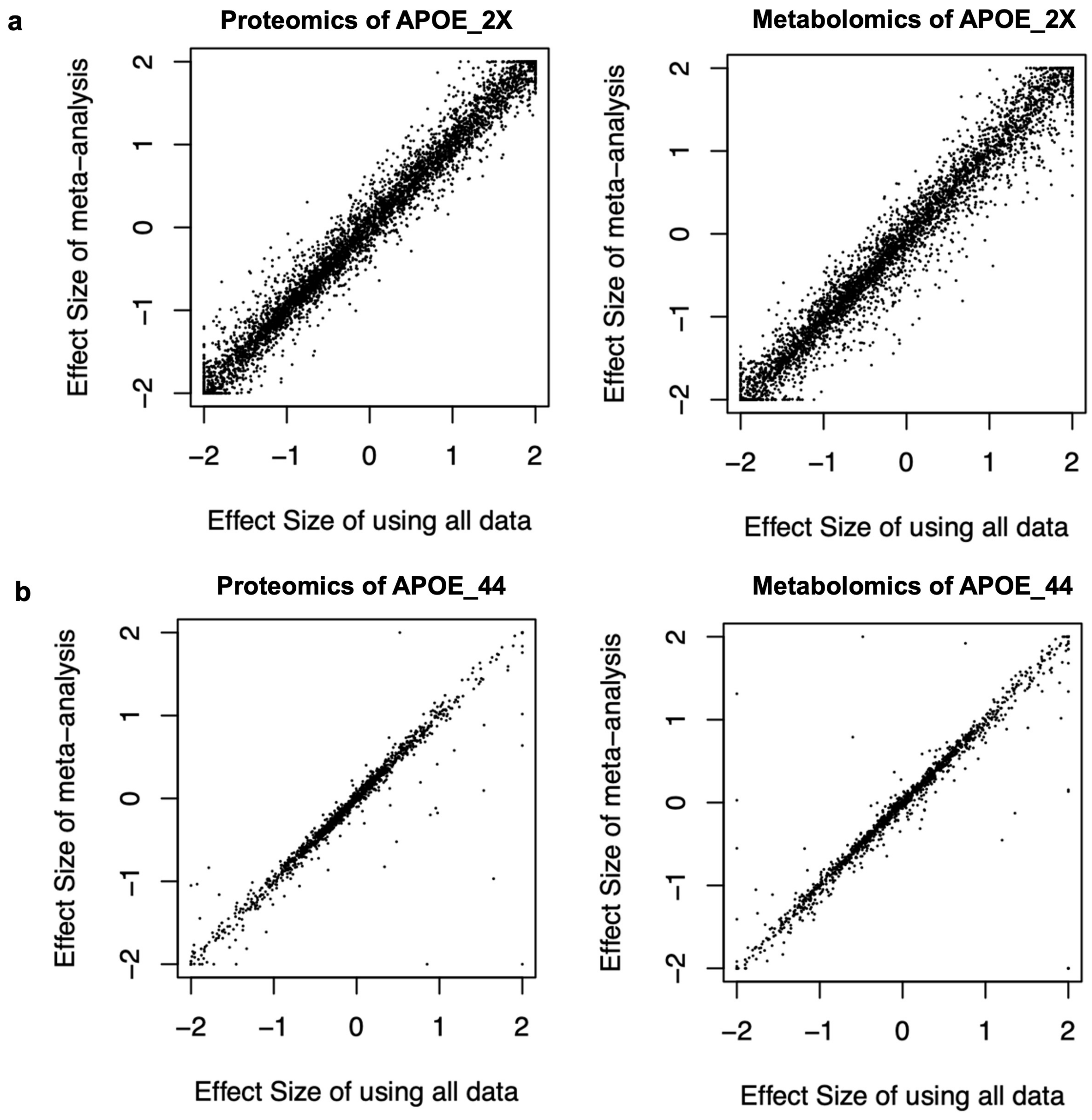

Figure S7: Scatter Plots of Effect Sizes in *APOE*_33 and *APOE*_34 Genotypes Comparing with Global Neurodegeneration Proteomics Consortium (GNPC), IADRC, Bio-Hermes and Stanford SomaScan Datasets.

a-d, Scatter plots of effect sizes (x-axis) for differentially expressed proteins in the *APOE*_33 group versus effect sizes (y-axis) from four external datasets: (a) GNPC, (b) IADRC, (c) Bio-Hermes, and (d) Stanford SomaScan. The number of overlapping aptamers is 468, 438, 468, and 468, respectively, with 450 (96%), 419 (96%), 437 (93%), and 446 (95%) falling within the 95% confidence interval. **e-h**, Corresponding plots for the *APOE*_34 group: (e) GNPC, (f) IADRC, (g) Bio-Hermes, and (h) Stanford SomaScan. The number of overlapping aptamers is 73, 67, 73, and 73, with 71 (97%), 62 (93%), 68 (93%), and 68 (93%) within the 95% confidence interval. **i-j**, Comparisons with the combined meta-analysis for (i) *APOE*_33 and (j) *APOE*_34, with 468 and 73 overlapping aptamers, of which 446 (95%) and 72 (99%) fall within the 95% confidence interval. Red dots indicate proteins within the 95% confidence interval defined by parallel dashed lines around the regression line. Grey dots represent proteins outside this interval. The middle line is the regression line.

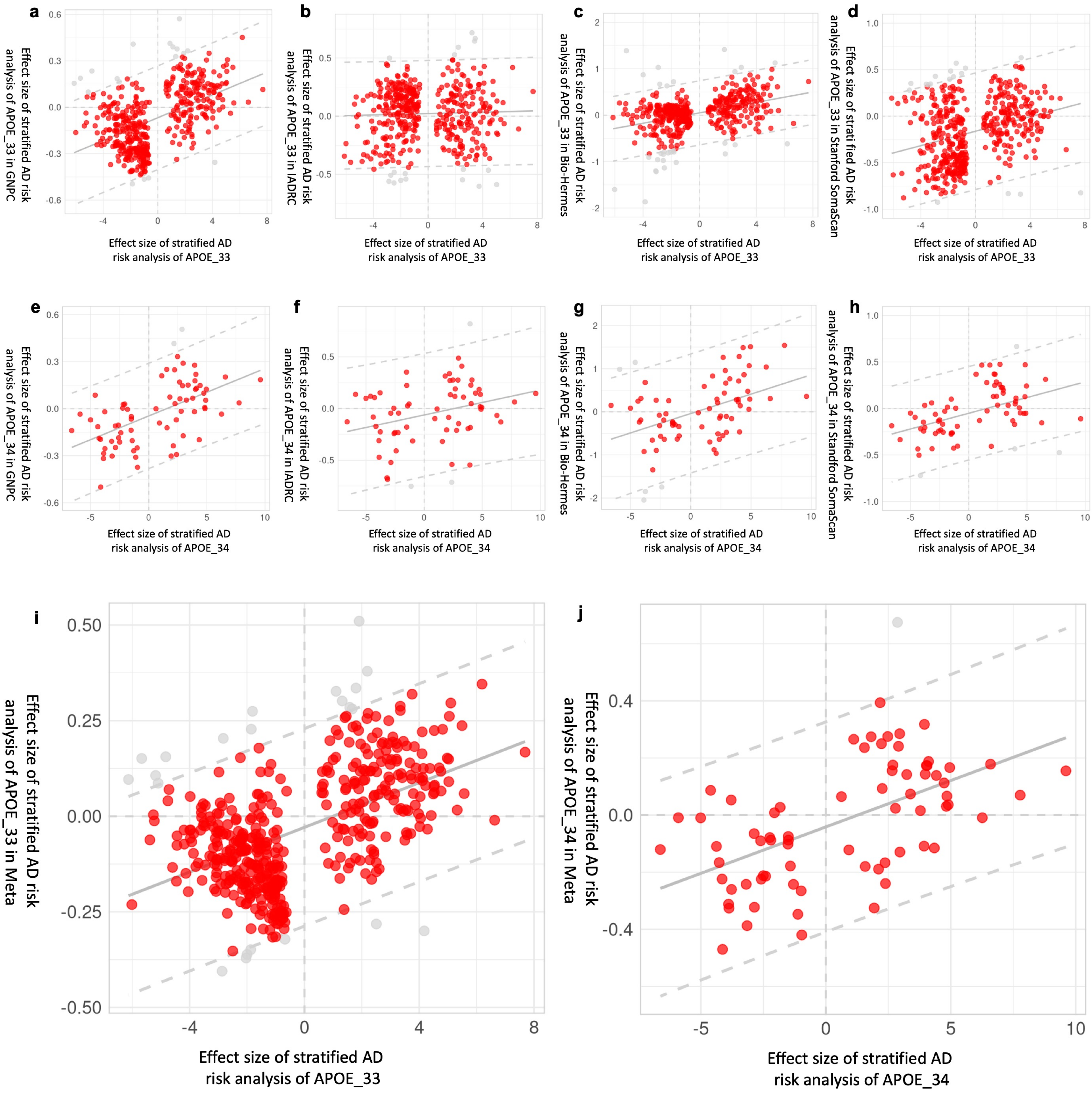

Figure S8: Scatter Plots of Effect Sizes in *APOE*_33 and *APOE*_34 Genotypes Comparing with UKBioBank (UKBB) and Stanford Olink Datasets.

a,c, Scatter plots of effect size (x-axis) of the differentially expressed proteins in (a) *APOE*_33 and (c) *APOE*_34 versus effect size (y-axis) of the proteins in UKBB dataset. There are 150 and 31 aptamers in *APOE*_33 and *APOE*_34; and 139 (93%) and 30 (97%) aptamers are within the 95% confidence interval. **b,d,** Scatter plots of effect size (x-axis) of the differentially expressed proteins in (b) *APOE*_33 and (d) *APOE*_34 versus effect size (y-axis) of the proteins in Stanford Olink dataset. There are 147 and 31 proteins in *APOE*_33 and *APOE*_34 that are also measured in Stanford Olink; and 136 (93%) and 30 (97%) proteins are within the 95% confidence interval. **e, f,** Scatter plots of effect size of the differentially expressed proteins in *APOE*_33 (e) and *APOE*_34 (f) versus the meta-analysis results combining UKBB and Stanford Olink datasets. There are 150 and 31 aptamers in *APOE*_33 and *APOE*_34; and 139 (93%) and 30 (97%) aptamers are within the 95% confidence interval. Red dots indicate proteins within the 95% confidence interval defined by parallel dashed lines around the regression line. Grey dots represent proteins outside this interval. The middle line is the regression line.

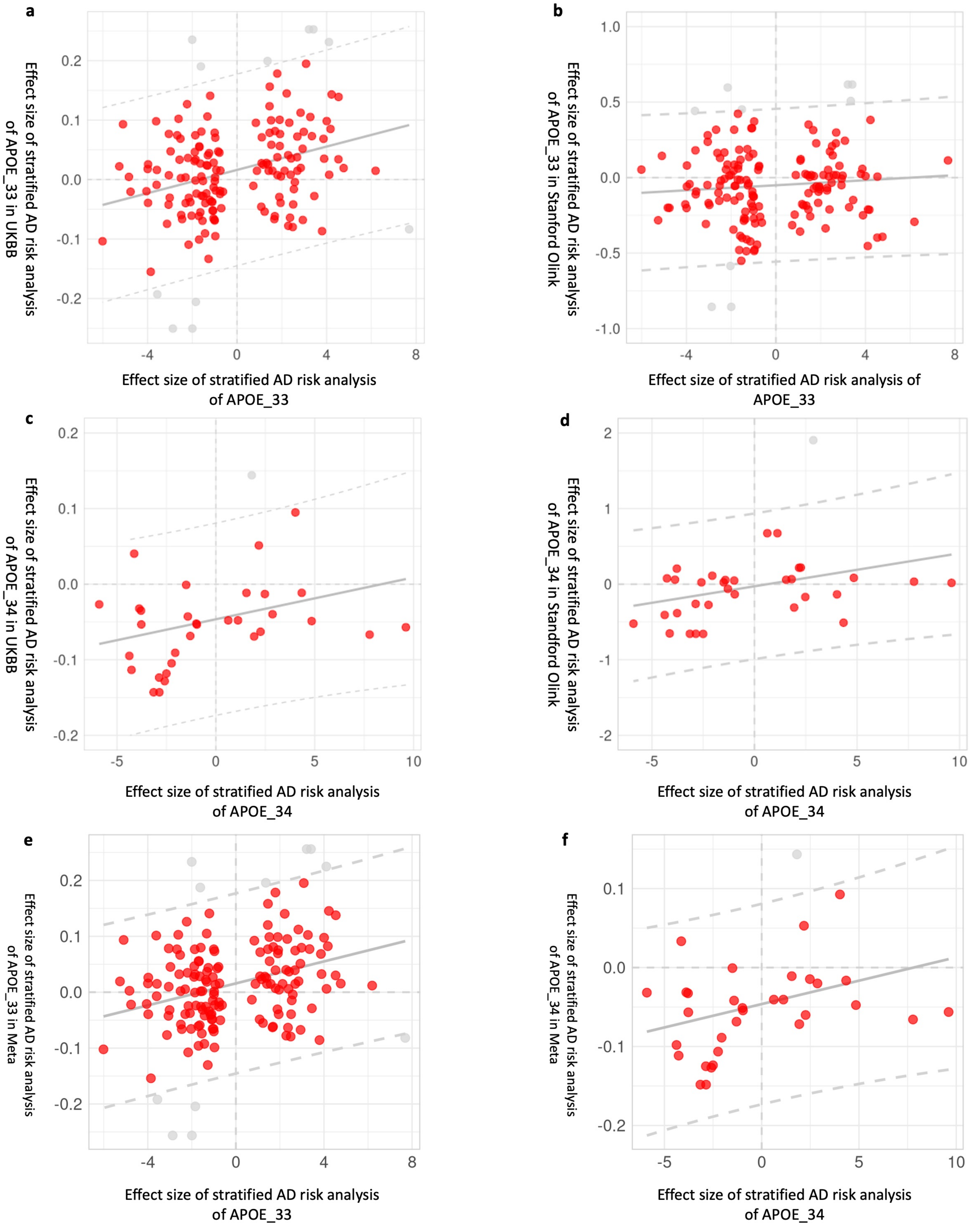

Figure S9: Scattering plots of effect size of selected proteins in stratified *APOE*_33 and *APOE*_34 and non-stratified analysis.

Scattering plots of effect size of selected proteins in stratified AD risk analysis in *APOE*_33 (468 aptamers) and *APOE*_34 (73 aptamers) and proteins selected in the non-stratified AD risk analysis (456 aptamers). Red points indicate proteins significant in both analyses, green points those significant only in APOE-stratified analyses, and blue points those significant only in non-APOE-stratified analyses.In the scattering plot of *APOE*_33, there are total 821 aptamers and 103 common aptamers. In the scattering plot of *APOE*_34, there are total 502 aptamers and 27 common aptamers.

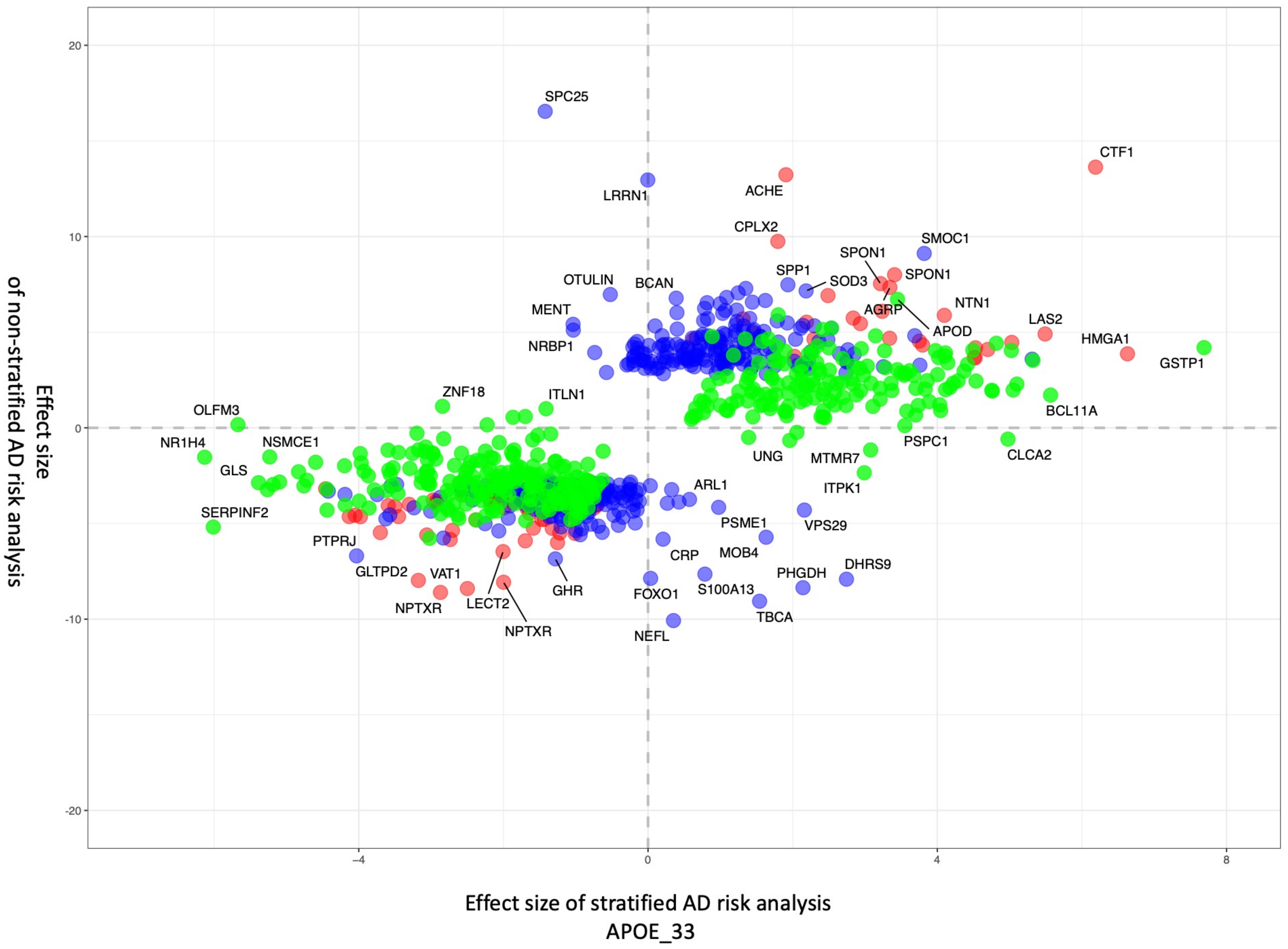

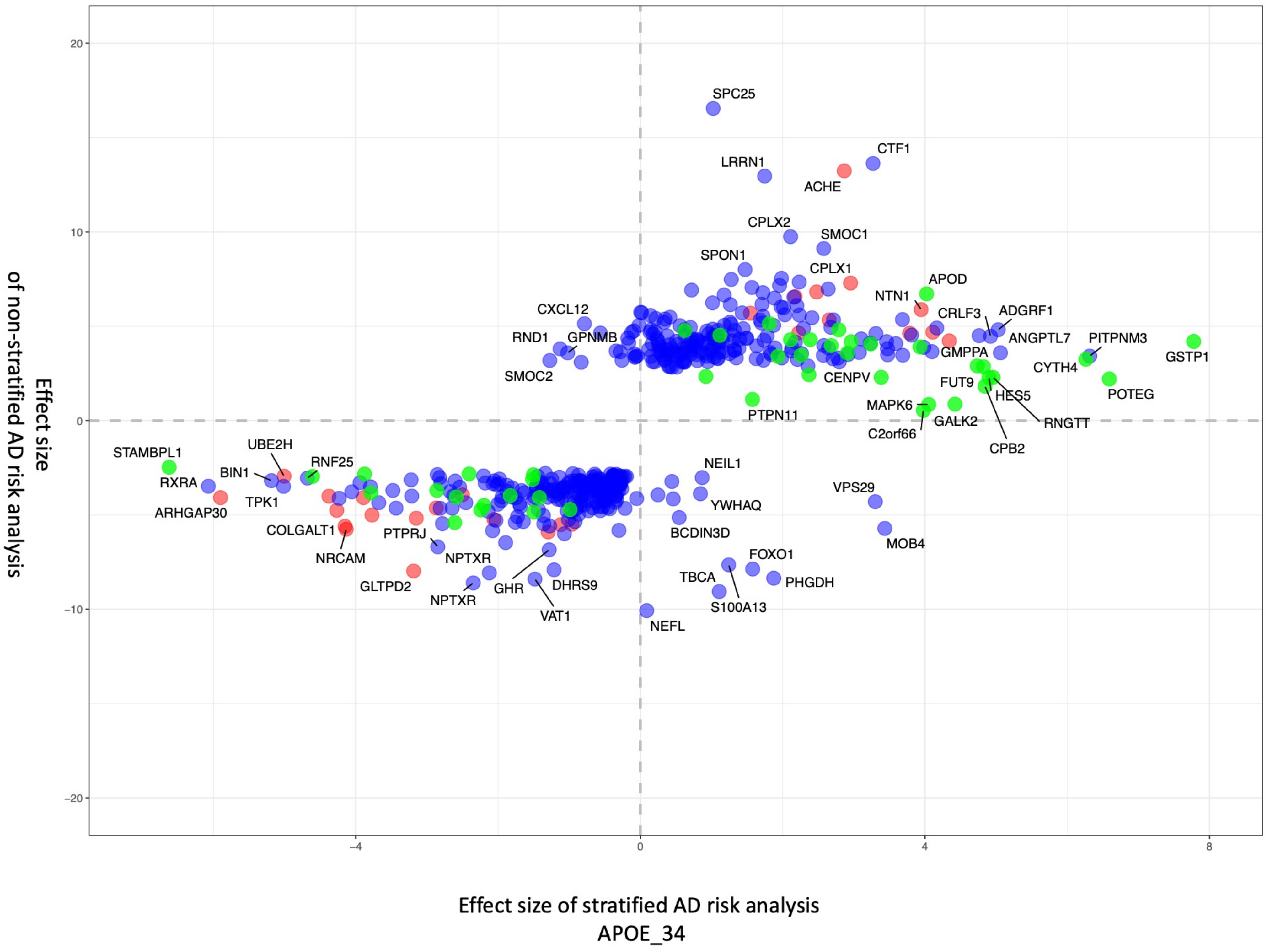

Figure S10: Enriched gene ontology (GO) terms in *APOE*_33, *APOE*_34 and *APOE*_4x genotypes.

The association network of up-regulated proteins and selected enriched GO terms of *APOE*_33 (**a**), *APOE*_34 (**b**) and *APOE*_4x (**c**). The association network of down-regulated proteins and enriched GO terms of *APOE*_33 (**d**), *APOE*_34 (**e**) and *APOE*_4x (**f**). Blue and red circle nodes represent down- and up-regulated proteins respectively. The green square nodes represent GO terms.

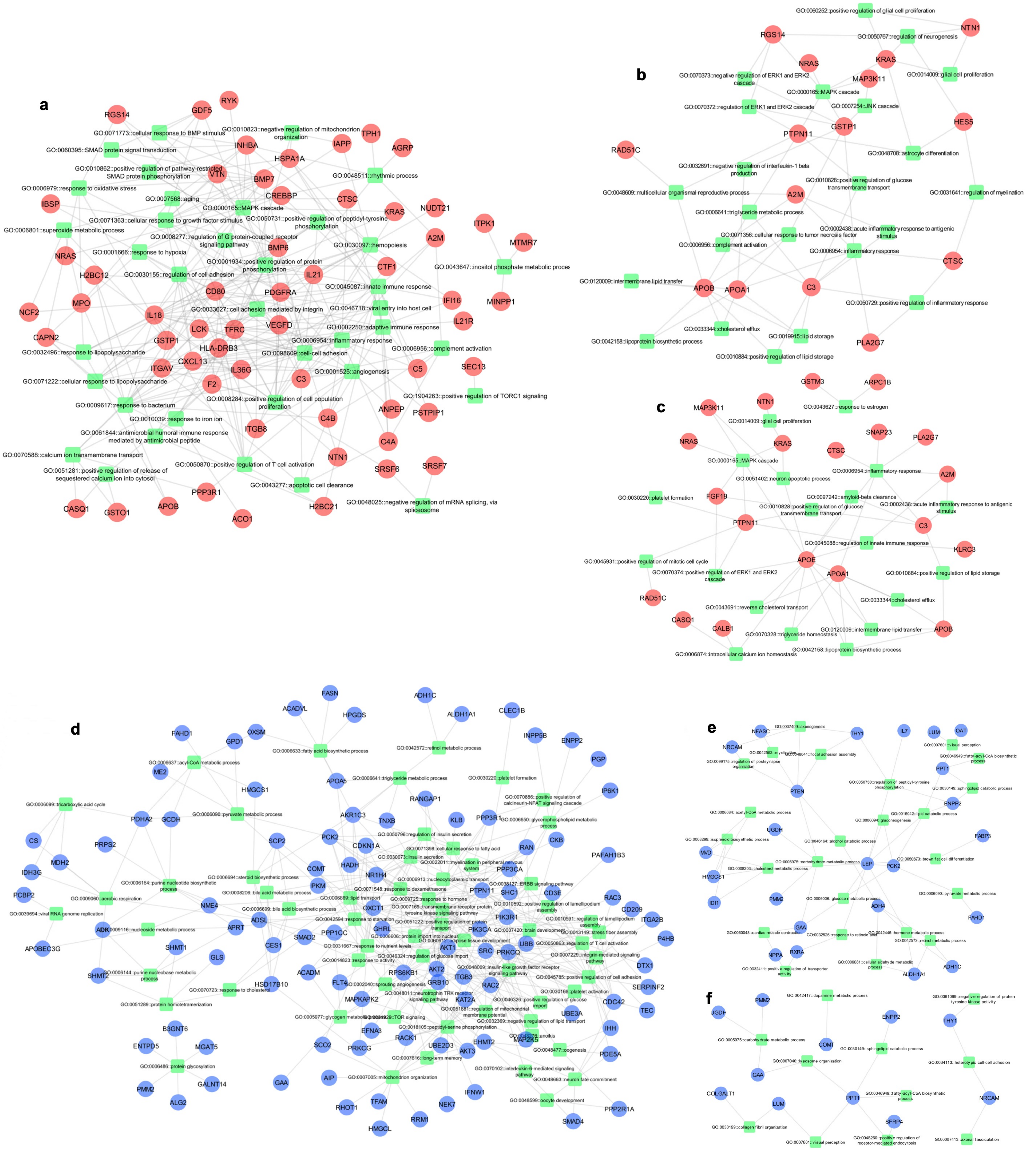

Figure S11: Heatmap plots of effect size values across six *APOE* genotypes of common proteomic and metabolomic biomarkers in *APOE*_33, *APOE*_34.

a, The heatmap plot of 36 aptamers (15 down- and 21 up-regulated) in *APOE*_33 and *APOE*_34.
b, The heatmap plot of 32 metabolites (18 down- and 14 up-regulated) in *APOE*_33 and *APOE*_34.
Each row represents one of the 6 APOE genotypes: 2x, 24, 33, 34, 44, 4x.

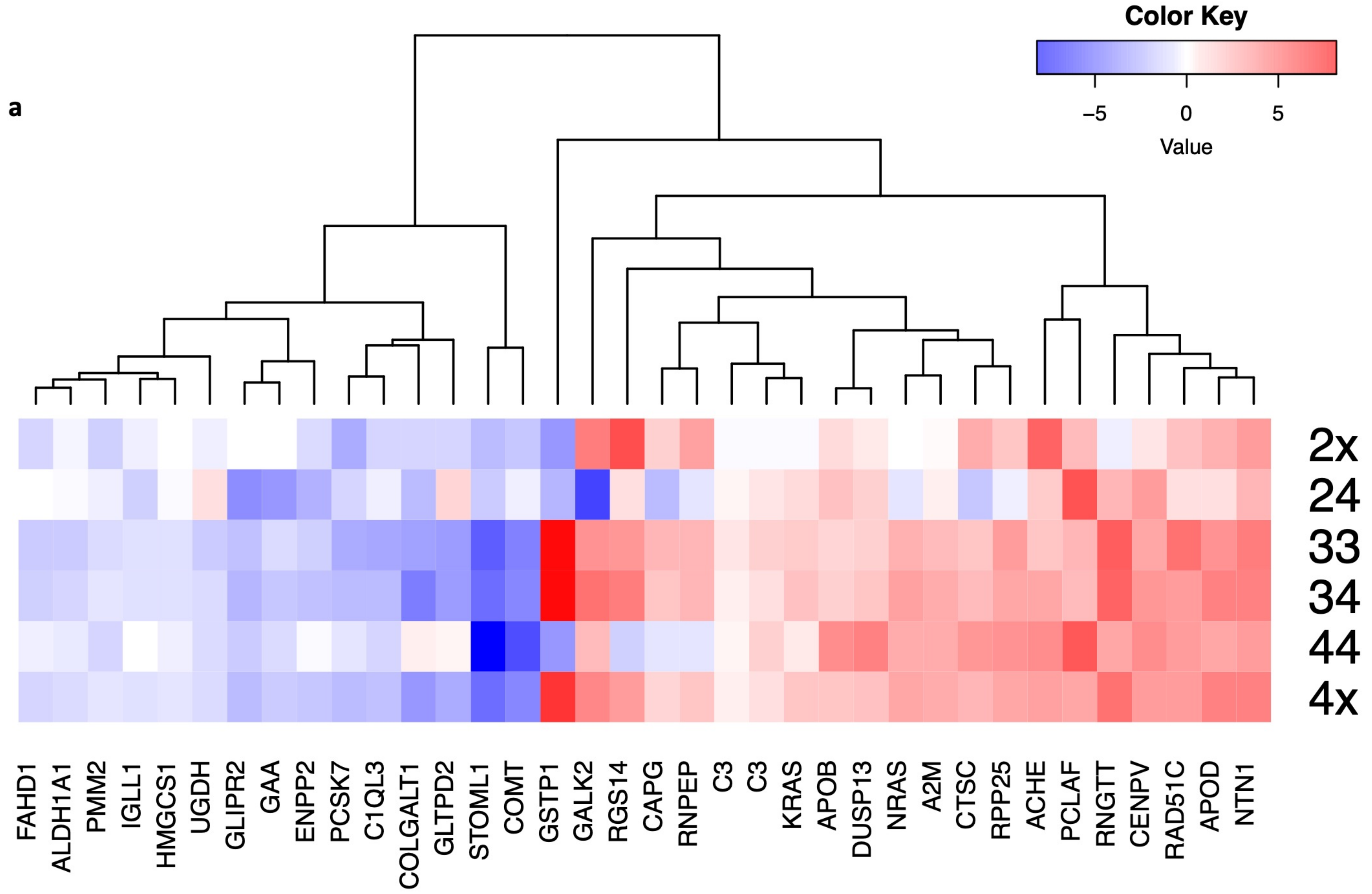

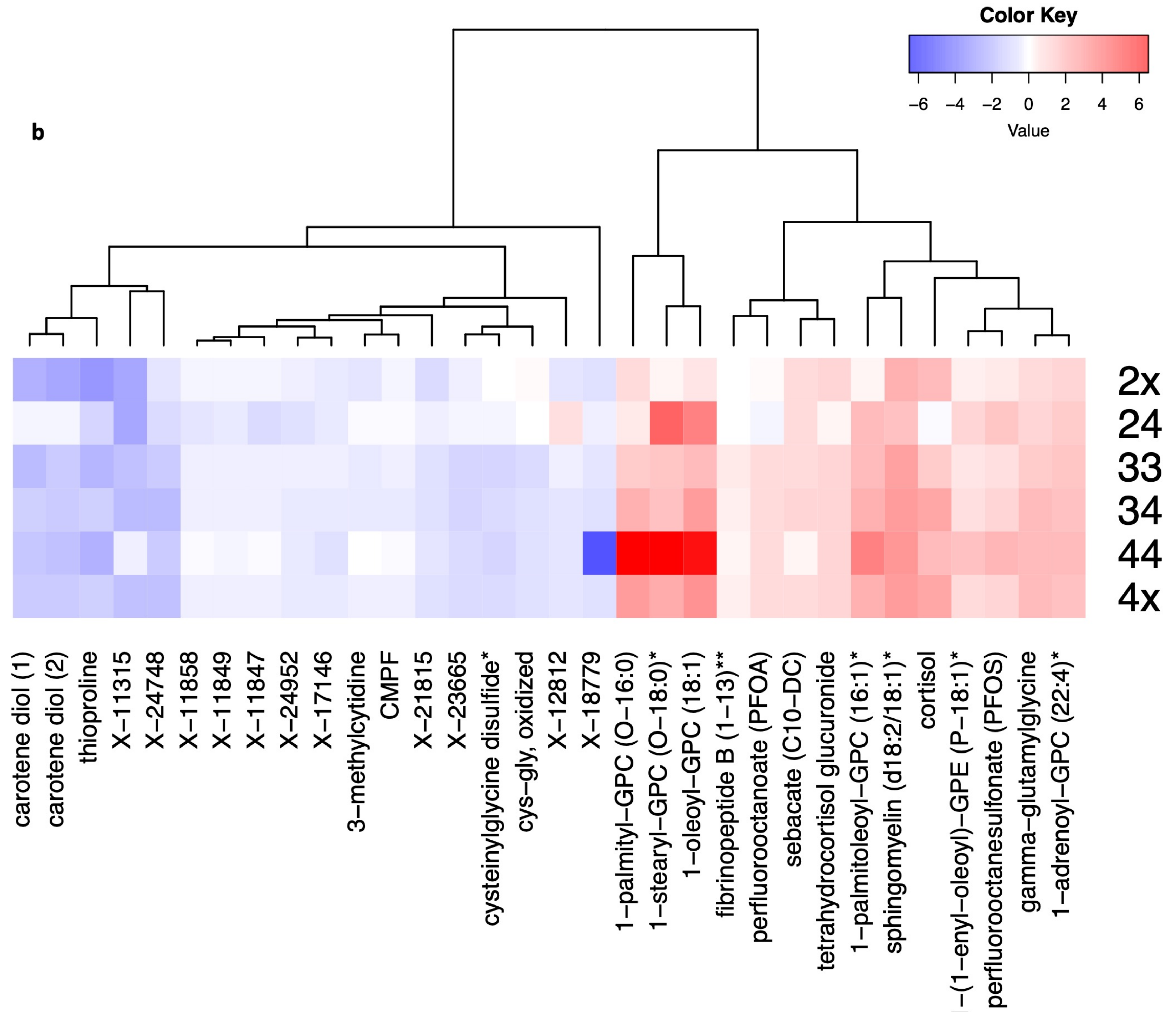

Figure S12: Heatmap plots of effect size values across six *APOE* genotypes of common proteomic and metabolomic biomarkers in *APOE*_33, *APOE*_4x.

a, The heatmap plot of 48 aptamers (22 down and 26 up-regulated) in *APOE*_33 and *APOE*_4x.
b, The heatmap plot of 39 metabolites (22 down and 17 up-regulated) in *APOE*_33 and *APOE*_34.
Each row represents one of the 6 APOE genotypes: 2x, 24, 33, 34, 44, 4x.

a

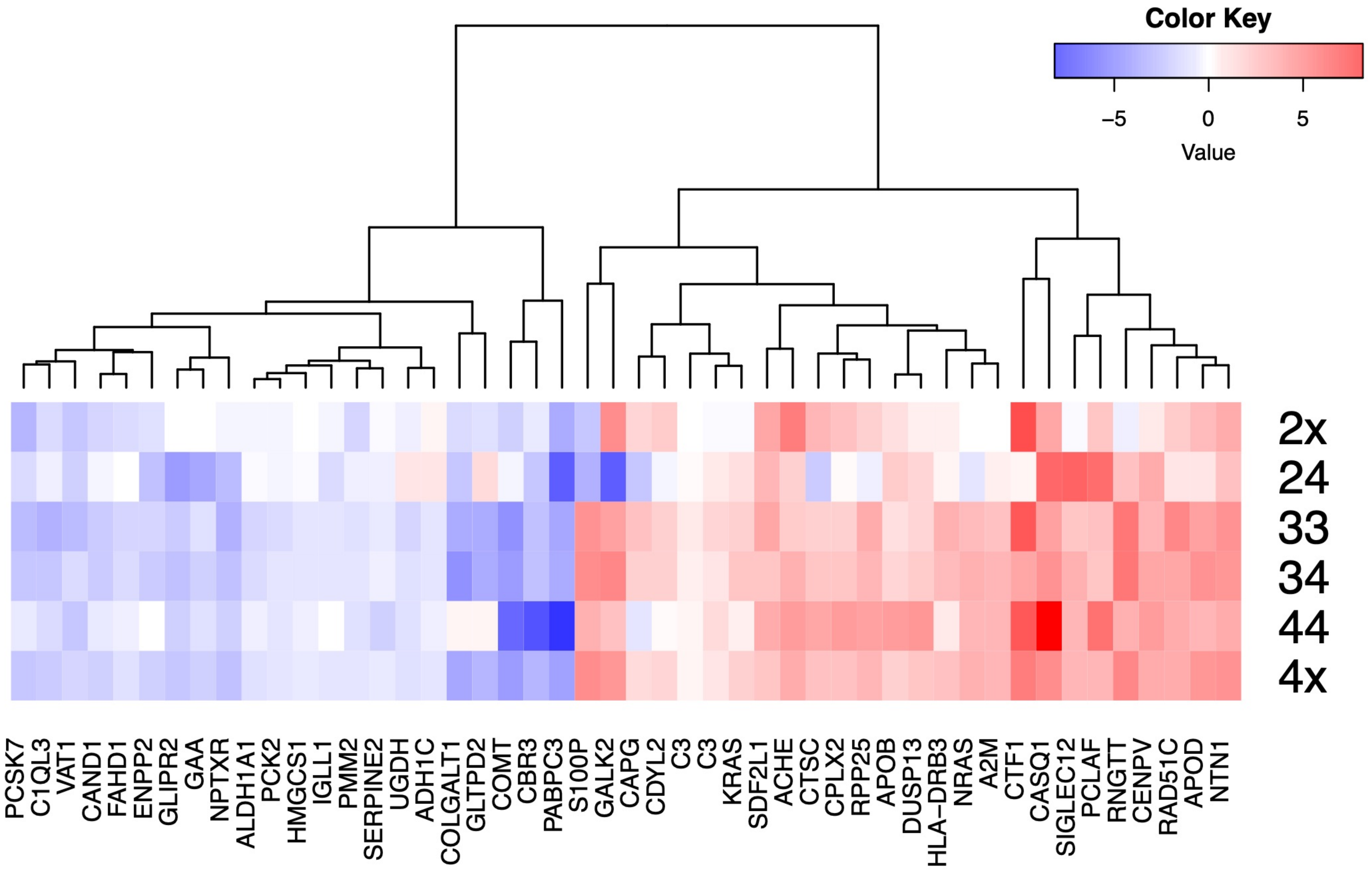

b

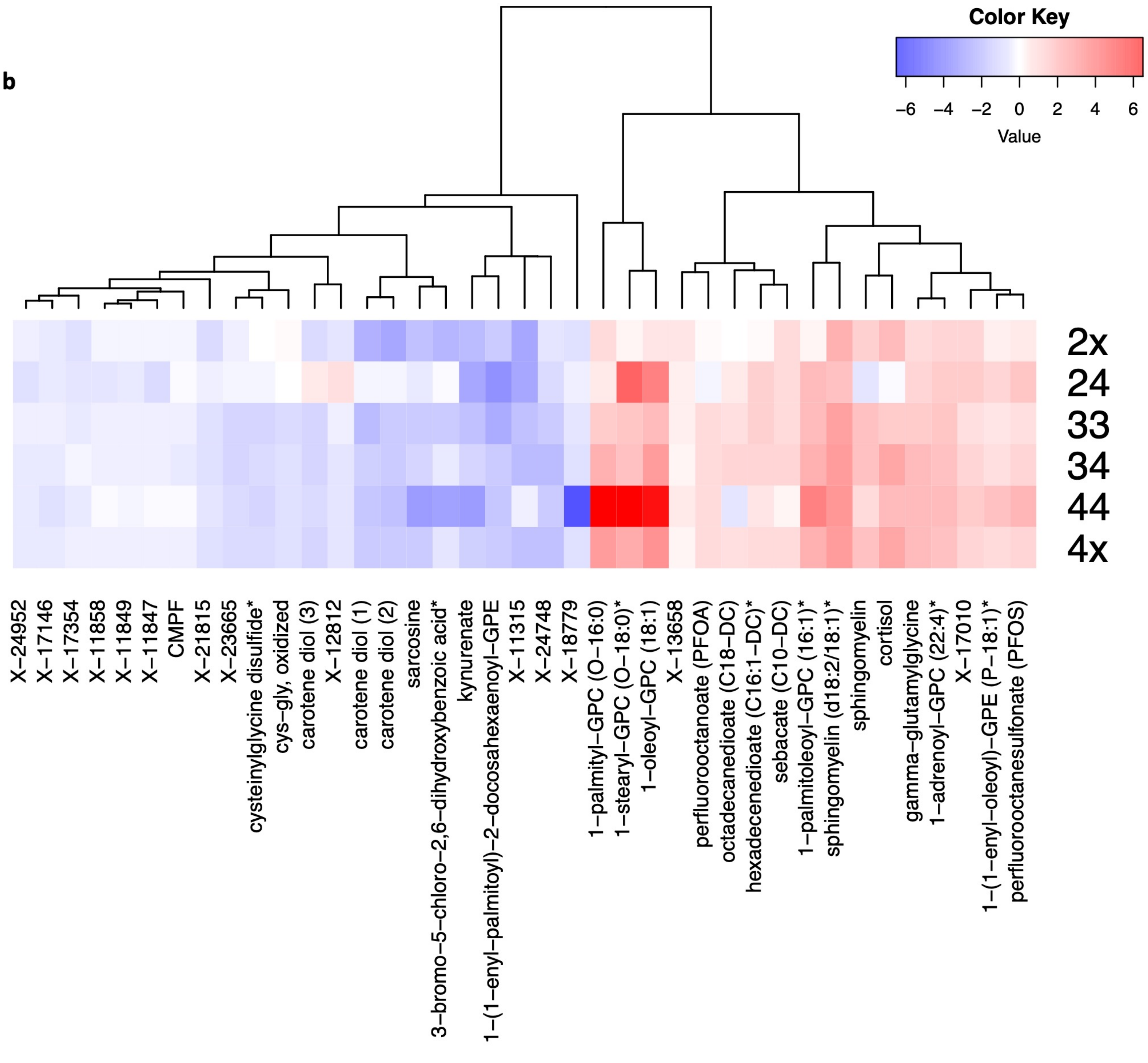

Figure S13: Signaling networks of consistent-up (A), consistent-down (B), Q1 (C), Q3 (D), Q2 (E), Q4 (F) generated by using the prize-collecting Steiner forest (PCSF) model.
The green circle nodes represent the given selected DEPs and the blue triangle nodes represent the linking proteins identified by the PCSF model to link the given proteins into signaling networks.

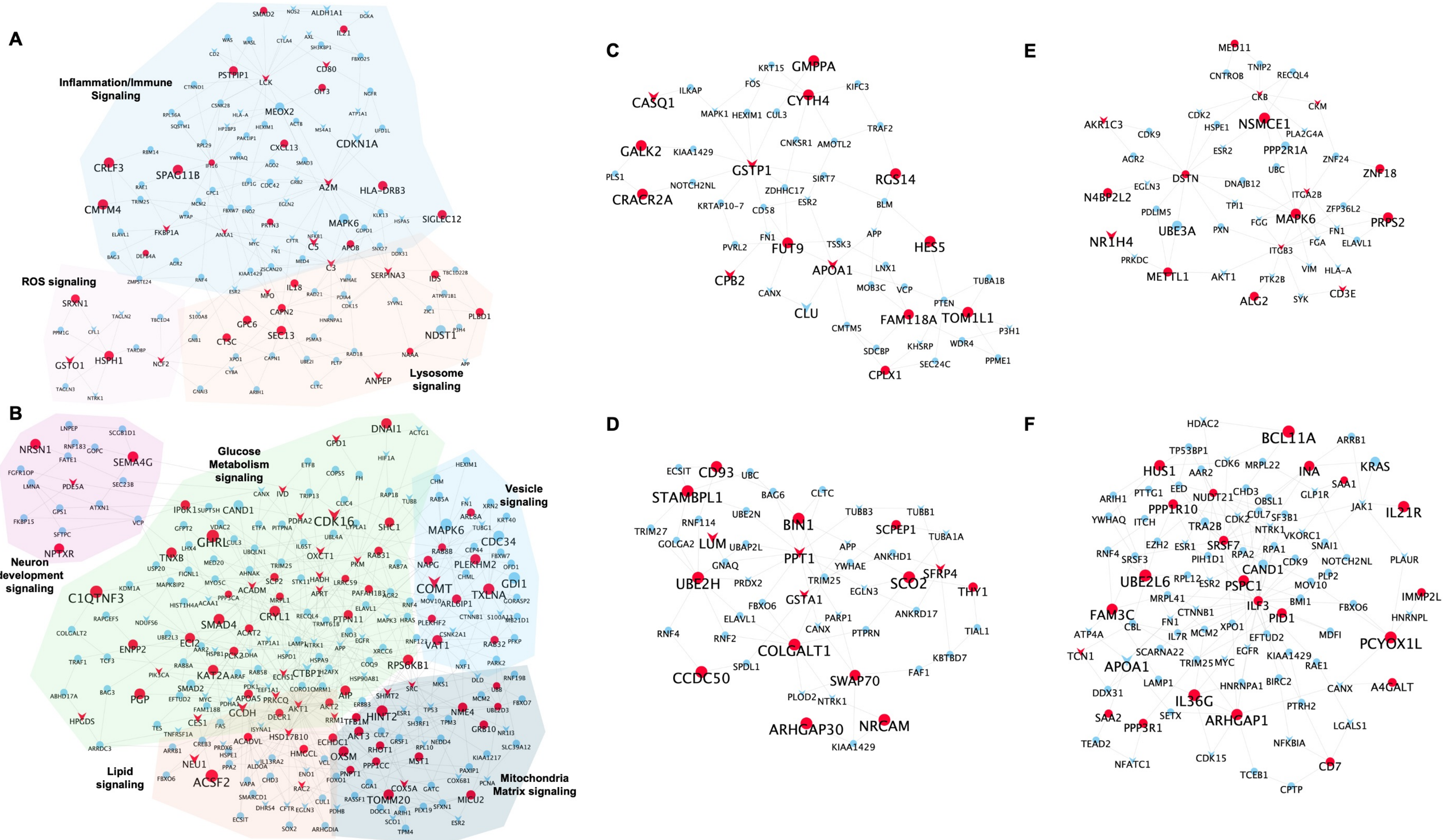

Figure S14. Brain Cell-type enrichment of differentially expressed proteins.

Log_2 fold enrichment of DEPs across brain-specific cell types for the proteins associated with AD in each subgroup. Consistent-Up **(a)**, Consistent-Down **(b)**, Q1 **(c)**, Q2 **(d)**, Q3 **(e)** and Q4**(f)**.

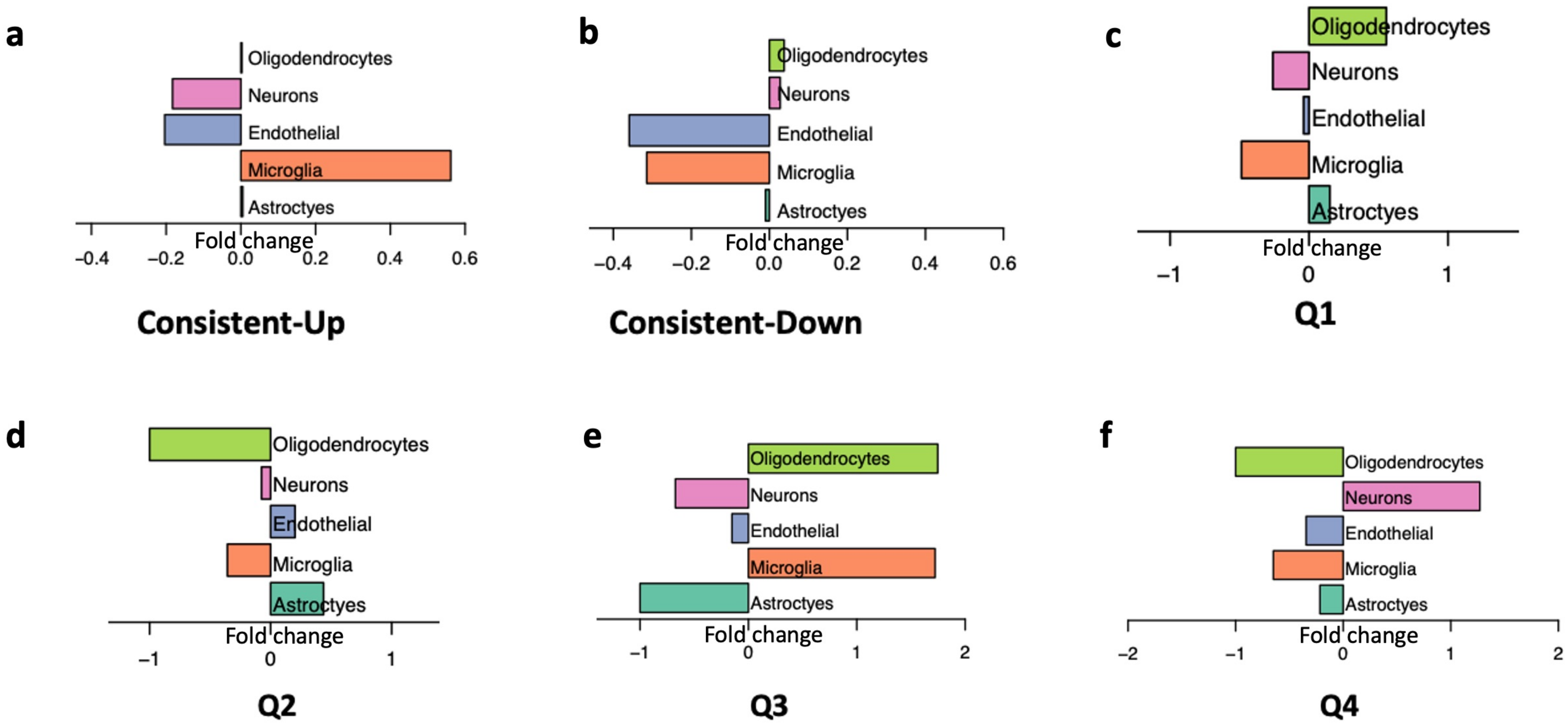

Figure S15. Druggable targets and drug repositioning.

(a) The 88 drug target proteins derived from DrugBank are highlighted with red color on the signaling network of the 494 differentially expressed proteins selected in *APOE*_33 or *APOE*_34. **(b)** The 14 categories of 34 top-ranked booster drugs (that can potentially activate the down-regulated proteins in both *APOE*_33 and *APOE*_34) derived using connectivity map (CMAP). **(c)** The 19 categories of 47 top-ranked inhibitor drugs (that can potentially inhibit the up-regulated proteins in both *APOE*_33 and *APOE*_34) derived using connectivity map (CMAP). **(d)** The drugs that can potentially activate the AKT signaling via the negative signaling regulation. The PIK3CA, PIK3R1, AKT1, AKT2, and AKT3 proteins are all downregulated in both *APOE*_33 and *APOE*_34.

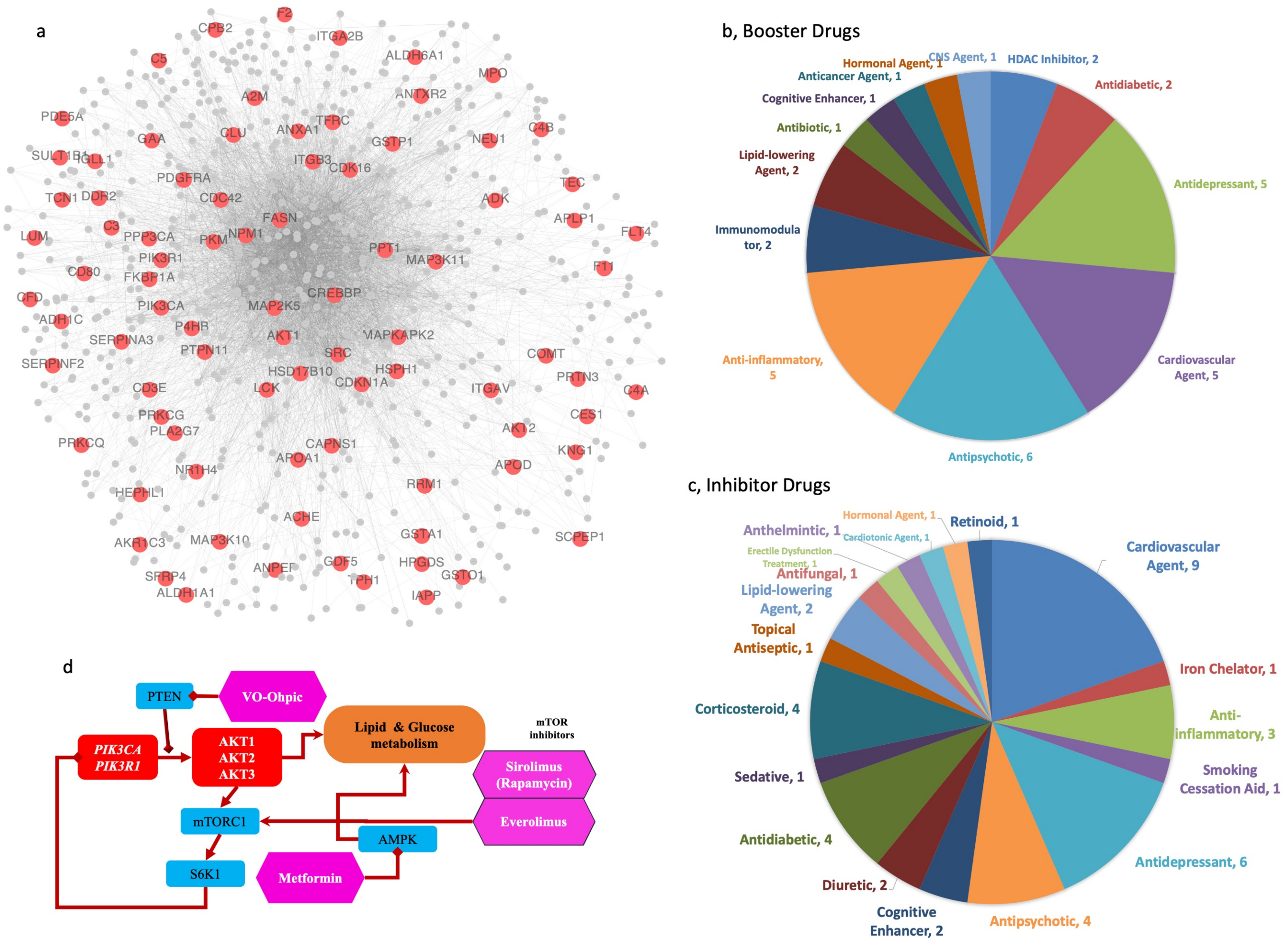

Supplement: Supplementary file 1 — Supporting File: advs74237‐sup‐0001‐SuppMat.pdf. [file ADVS-13-e13872-s001.pdf]
